# White and gray brain matter activity during pain in fibromyaliga patients and healthy controls

**DOI:** 10.1093/braincomms/fcaf409

**Published:** 2025-10-17

**Authors:** Peter Fransson, Silvia Fanton, Karolina af Ekenstam, Monika Löfgren, Jörgen Rosen, Karin Jensen, Eva Kosek

**Affiliations:** Department of Clinical Neuroscience, Karolinska Institutet, Stockholm, SE-171 76, Sweden; Department of Radiology, Massachusetts General Hospital, Harvard Medical School, Boston, MA 02115, USA; Athinoula A. Martinos Center for Biomedical Imaging, Massachusetts General Hospital, Charlestown, MA 02129, USA; Department of Clinical Sciences, Karolinska Institutet, Danderyd University Hospital and Department of Rehabilitation Medicine, Danderyd University Hospital, Stockholm, SE-182 88, Sweden; Department of Clinical Sciences, Karolinska Institutet, Danderyd University Hospital and Department of Rehabilitation Medicine, Danderyd University Hospital, Stockholm, SE-182 88, Sweden; Department of Clinical Neuroscience, Karolinska Institutet, Stockholm, SE-171 76, Sweden; Department of Clinical Neuroscience, Karolinska Institutet, Stockholm, SE-171 76, Sweden; Department of Clinical Neuroscience, Karolinska Institutet, Stockholm, SE-171 76, Sweden; Clinical Pain Research, Department of Surgical Sciences, Uppsala University, Uppsala, SE-751 85, Sweden

**Keywords:** white matter functional MRI, brain, chronic pain, fibromyalgia

## Abstract

Functional neuroimaging studies have shown that a set of cortical brain regions is typically engaged during noxious stimulation and pain perception in humans. Other studies have focused on cortical brain activation patterns in patients suffering from chronic pain conditions such as fibromyalgia. Recent work has shown that functional MRI signals are also present in white matter. In this study, we present an analysis of white and grey matter functional MRI activation during a block-designed pain stimulus task in cohorts of fibromyalgia patients and healthy controls. Task data and resting-state functional MRI data were collected from female fibromyalgia patients (*N* = 54) and controls (*N* = 56). Pain-stimulation task-based functional MRI included noxious pressure of the left shin as well as an appraisal of pain intensity. White and grey matter brain activities related to pain stimulation were analysed in 29 white matter and 200 grey matter regions-of-interest using time-locked activation analysis. In the healthy cohort, we present evidence of functional MRI brain activity in response to processing painful stimuli for a large majority of cerebral white fibre tracts investigated. In contrast, white matter functional MRI activity in the fibromyalgia cohort was limited to the contralateral posterior and anterior limb of the internal capsule as well as the bilateral cerebral peduncles. Pain stimulation and appraisal of pain intensity also resulted in widespread time-locked functional MRI activity in grey matter, such as visual, somatomotor, fronto-parietal attention and frontal networks as well as in the insular cortex. We have shown that white matter functional MRI signal changes localized to thalamocortical projection fibres, which are attributed to sensory and motor neuronal processing, are related to the perception and appraisal of pain in both fibromyalgia patients and healthy controls. Our results suggest that functional MRI of white matter projection fibres provide additional information that goes beyond task-induced functional MRI responses in cerebral grey matter, with a potential to become clinically useful in the future.

## Teaser

Fransson *et al*. collected pain task functional MRI data in grey and white brain matter regions in a cohort of healthy controls and fibromyalgia patients. White matter functional MRI activity was localized to thalamocortical projection fibres related to perception and appraisal of pain in both cohorts.

## Graphical Abstract

**Graphical Abstract:**
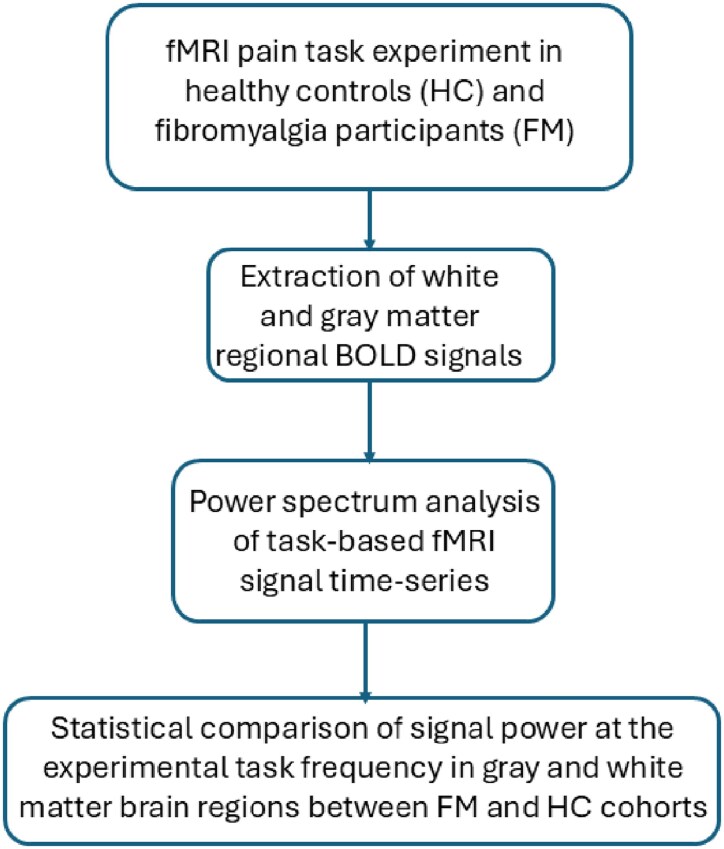

## Introduction

Cerebral responses during pain stimulation have been studied in humans using non-invasive functional neuroimaging methods for almost four decades.^1^ Early studies using PET, and later blood oxygenation level–dependent (BOLD) functional MRI (fMRI) consistently showed that a certain set of cortical brain regions is typically engaged during noxious stimulation and pain perception in humans.^2^ In parallel, neuroimaging studies have focused on elucidating putative changes in brain activity in patients suffering from chronic pain, e.g. fibromyalgia (FM) and rheumatoid arthritis.^3,4^

Over 20% of the world population experience chronic pain at any given time point and some of the most common pain conditions, like non-specific low-back pain and FM, are regarded as nociplastic pain conditions,^5^ with nociplastic pain being a result of neuronal plasticity, including sensitization in afferent nociceptive tracts as well as top–down amplification and disinhibition of nociceptive signals. FM is a common pain syndrome that affects 2–4% of the general population, whereof 80% being women. Core symptoms of FM include persistent widespread pain, tenderness, fatigue and cognitive impairments.^6^ The treatment options are scarce, as traditional analgesics are generally not effective, and multidisciplinary pain management has yielded varied results.

Previous studies from our group have shown differences in brain structure and function in FM compared to healthy controls (HCs)^7,8^ and evidence suggest that FM and other forms of nociplastic pain are associated with neuroinflammatory processes in the spinal cord and brain, affecting the level of activity of glial cells.^9,10^ Recent studies have targeted the cause of neuroinflammation in FM by studying the uptake of PET radiolabeled ligands that bind to the translocator protein (TSPO), which is upregulated in activated microglia and astroglia. Increased levels of TSPO have been observed in the primary sensorimotor cortices, frontal and parietal.^11-13^ However, none of these studies on chronic pain have examined glial activity in fibre tracts in white matter (WM), apart from the Albrecht *et al*. study that found no change in WM TSPO uptake when all WM voxels in each hemisphere were collapsed into a single region-of-interest.

Since its inception,^14^ BOLD fMRI imaging of the human brain has been focused on grey matter (GM), with little attention given to BOLD signal changes in WM. Accordingly, signal changes in WM are often treated as noise and discarded in imaging processing pipelines. However, recent experiments have shown that task-related BOLD signal changes are present also in WM, although displaying a relatively smaller amplitude and higher variability in response profiles.^15-20^

Given the potential to detect WM BOLD signal changes and the potential importance of WM integrity in chronic pain disorders, we collected pain-stimulation and resting-state fMRI data from FM patients and HC. To assess changes in brain activity in both GM and WM, we performed a time-locked analysis of BOLD signal responses in both cohorts. We aimed to address the following questions: Are BOLD signal changes in WM fibre tracts sensitive to the perception and appraisal of pain and can these changes be reliably detected within and across cohorts? If so, are there systematic differences in WM BOLD activity between FM and HC? Are time-locked BOLD signal profiles in GM different between cohorts?

## Methods

### Patients

In total, 86 controls and 87 patients with FM were enrolled in the study. MR imaging was available from 53 FM and 56 HC. Patients were recruited via advertisement, and a full list of inclusion and exclusion criteria is given in the Supplementary material. Patients gave written informed consent in accordance with the Declaration of Helsinki before participating in the study. Patients were remunerated for their time, and the study was approved by the local ethical authority board (ethics permit: 2019/06161). The study was pre-registered at clinicaltrials.gov (ID: NCT05815381, but the investigation of WM BOLD signals changes in response to pain stimulation was not a part of the pre-registration.

### Questionnaires and behavioural assessments

The participant characteristics are presented in Table S1. Patients rated their pain intensity at the day of examination as well as the average, minimal and maximal pain intensity during the past week on a 100 mm visual analogue scale (VAS) anchored by the words ‘no pain’ and ‘worst imaginable pain’. The widespread pain index (WPI) corresponding to the number of painful body regions (0–19), as well as the symptom severity score (SSS; 0–12) was determined for all individuals. WPI and SSS form part of the 2016 revision of the 2010/2011 diagnostic criteria for FM.^21^ The impact of FM was assessed by the Fibromyalgia Impact Questionnaire (FIQ), ranging from 0 to 100, where 100 represents maximal negative impact.^22^

Sensitivity to pressure was assessed by an experimenter using a handheld algometer (Somedic Sales AB, Hörby, Sweden) with a probe area of 1 cm^2^ and a rate of pressure increase at approximately 50 kPa/s, using a digital screen for a visual feed-back.^23^ Patients were instructed to press a push-button as soon as the increasing pressure went from a pressure sensation into a painful sensation (pressure pain threshold, PPT). To assess sensitivity to suprathreshold pain stimuli, patients were instructed to press the push-button first when they would rate the pain as a 4 (moderate to strong pain) or 7 (very strong pain), respectively, using Borg's category ratio (CR)-10 scale,^24^ where 0 = no pain and 10 = extremely strong pain. Pressure algometry was performed at the left forearm and the left thigh.

### Image acquisition

Structural and fMRI as well as magnetic resonance spectroscopy (MRS) data were acquired on a General Electric MR750 3 Tesla whole body scanner using an eight-channel head-coil. Anatomical imaging (3D, T_1_-weighted image, 1 mm^3^ voxel resolution) was followed by single voxel MRS of the right posterior and anterior insula regions. The MRS data will not be further analysed and discussed in the present study. Patients underwent two pain-stimulation BOLD fMRI runs as well as a resting-state run (T_2_*-weighted imaging, number of slices = 42, slice thickness = 3 mm, slice gap = 0.5 mm, TR = 2.2 s, TE = 30 ms, flip angle = 70°, field-of-view = 220 × 220 mm, 72 × 72 voxel matrix, 3 × 3 × 3.5 mm^3^ voxel size). The pain-stimulation runs included 180 image volumes whereas the resting-state run included 274 image volumes. During the resting-state run, patients were instructed to rate their perception of on-going clinical pain (FM) or degree of physical unpleasantness (HC) using a trackball device. The unpleasantness rating in the HC cohort was included for the sole purpose of making the pain task paradigms as similar as possible across cohorts in terms of sensory and visual input as well as motor execution. The resting-state run was followed by the two pain-stimulation runs that included pressure to patients’ left shin.

Pressure stimuli during fMRI scanning were delivered by means of an automated, computer-controlled, pressure device with a 1 cm^2^ probe (Automated Pressure Applicator, Somedic, Hörby). The first pain-stimulation scan included pressures of 150 kPa applied to the left shin, whereas in the second run the pressure was increased to 300 kPa. After each painful stimulus, the patients rated the perceived pain intensity.

The 300 kPa pressure stimulus was well-tolerated by all HC but was regarded too painful for six FM patients. In this study, only the data from the 300 kPa scan was analysed and presented as it was deemed painful enough among HC, yet still tolerable for most FM patients. The most common way to assess cerebral pain-related activation is to calibrate pressure stimuli individually to correspond to a predefined pain intensity.^7,10^ However, for reasons explained in the pre-registration (ID: NCT05815381), the present study used the same absolute pressure intensity for all patients.

### Design of the pain stimulation and appraisal of pain task paradigm

A schematic picture of a single block in the pain-stimulation paradigm is given in Fig. S1. Each block started with a jittered interstimulus interval (ISI1, range: 1.5–3.5 s, followed by a preset phase when the automated pressure device descended the probe to a fixed position 5 mm above the skin of the left shin, and a second jittered rest interval (ISI2, same jittering interval as for ISI1). Next, a forewarning (FF, 1 s), including an initial brief flicker at the intersection of a cross was given to prepare the patients for the upcoming pressure stimulus. The duration of each pressure to the participant’s left shin was set to 5 s. The termination of the pressure stimuli was succeeded by a rest period of 11 s, after which a visual presentation of the pain rating scale (0–100 mm VAS ranging from 0 = no pain to 100 = worst imaginable pain) appeared on the screen for 7 s and the patients could rate the pain intensity using the side buttons of a trackball. The control patients were instead of pain asked to rate their experienced level of unpleasantness on a 0–100 rating scale. A total of 10 blocks were presented during each of the two pain-stimulation runs.

### Resting-state fMRI scanning

During the entire 10-minute resting-state scan, patients were presented with a visual scale (0–100 mm VAS) that continuously showed their rating of clinical pain (FM) or physical unpleasantness (HC). Throughout the resting-state scan, patients could change their rating along the visual scale by using the trackball device. The initial idea of continuously recording the ratings of pain was to test our hypothesis that oscillatory changes in appraisal of pain during a resting-state run correlate with resting-state network connectivity. However, as shown in Fig. S2, most FM patients reported a marked increased level of perceived pain early on during the resting-state scan, reaching a high plateau after only 1–2 min, with very little or no change during the remainder of the run. Hence, due to the very small changes in perceived pain across time, we deemed it not meaningful to test this hypothesis. The appraised level of unpleasantness in HC throughout the resting-state run remained low as shown in Fig. S2.

### Image pre-processing

Functional resting-state and task data were preprocessed in the software package SPM12 (Wellcome Trust Centre for Neuroimaging). Raw fMRI images were realigned for motion correction (three rotational and three translational regressors), co-registered to the anatomical scan and normalized to the MNI template as implemented in SPM12. No spatial smoothing of the functional neuroimaging data was performed. The degree of residual head-movement in the data was assessed using the Framewise Displacement (FD) parameter.^25^ If the mean FD was larger than 0.5 mm during any scan, that participant was excluded from further analysis. These exclusion criteria resulted in discarded data from three FM and one HC participant that were thus not used in the analysis. Additionally, due to a technical error, data logs of task paradigm timing were missing for two patients (1 FM, 1 HC). Accordingly, time-locked analysis of pain task and resting-state fMRI data was performed in 41 FM and 54 HC patients. The inclusion of a global mean signal regression (GSR) step in the image pre-processing pipeline has for a long time been debated in the literature (see, e.g. Fox *et al*. and Potts *et al*.^26,27^). It has been argued that GSR has beneficial properties in terms of correcting for spurious signals related to residual effects from subject head-motion and respiratory pulsations.^28^ However, a recent study by Bolt *et al*. that employed large cohorts of simultaneous EEG and fMRI recordings has shown compelling experimental evidence that the global fMRI signal is intrinsically linked to the brain’s arousal response system, primarily the sympathetic nervous system.^29^ The findings by Bolt *et al*. have direct implications for the choice of including the GSR pre-processing step in the current study. It is likely that pain and experience of painful events will trigger the brain’s autonomic nervous system by increasing the degree of arousal, for example by priming the body for heightened sensory input and pain processing. With these considerations in mind, we refrained from using GSR, since this image pre-processing step would lead to any potential pain-related differences in brain’s arousal system between cohorts being regressed out from the data.

### Parcellation and definitions of regions-of-interest in WM and GM

Parcellation of WM tracts was performed using the ICBM (International Consortium of Brain Mapping) WM atlas.^30^ The ICBM parcellation scheme separates WM tracts into 48 separate regions-of-interest (ROI) (see Table S1 for a full description). However, the analysis of WM BOLD signal changes is sensitive to signal contributions from neighbouring GM voxels. To minimize the influence from GM, we employed a conservative masking approach so that only voxels in the ROIs that had a 95% or higher probability of residing in WM were included in the analysis. For the masking procedure, the WM probability map (‘white.nii’) supplied within the SPM package was applied to all ICBM WM ROIs. As a result, the number of accepted voxels in most WM ROIs was substantially reduced, and in some regions, no voxels at all survived the chosen threshold. After masking, 29 WM ROIs remained for the analysis of BOLD signal changes (see also Table S2 for details regarding the number of remaining voxels in each ROI). For parcellation of cortical GM, we used the Schaefer/Yeo 7-network 200 ROI parcellation scheme^31^ to define borders of ROIs in GM. The average BOLD signal from WM and GM ROIs were extracted from all subjects during the pain-stimulation task and resting-state and used for time-locked activation analysis.

### Analysis of time-locked activations

The shape and appearance of the BOLD haemodynamic response function (HRF) in GM is well documented.^32^ However, as shown in Li *et al.*^19^ the temporal profile of the HRF in WM varies considerably between regions and tasks, which renders the usage of a canonical form of the HRF function not optimal. To address this issue, we instead chose to investigate time-locked brain activation profiles^15,19^ in response to task blocks of perception and appraisal of pain (Fig. S1). Time-locked activation analysis takes advantage of the repetitive aspect of the blocked design. Our task included 10 blocks of identical pain stimuli and VAS ratings of pain with an average block length of 38.43 s (see also Figs S1 and S3). This corresponds to an average task frequency of 0.026 Hz. Thus, to investigate the presence of time-locked activation, we estimated the power spectrum for each ROI (using the periodogram method provided in the scipy library (version 1.11.1) in python (version 3.11), which computes the distribution of signal power per unit of frequency). The periodogram method in scipy computed the power amplitude at 91 separate frequency bins in the frequency span given by the sampling frequency (0–0.277 Hz). To determine if time-locked activity in each ROI was statistically significant at the task frequency, we compared the ROI signal power at the closest corresponding frequency bin (0.02525 Hz) for the pain-stimulation task data with corresponding value obtained from power spectrums computed from the resting-state scan.

Because the signal power at task frequency during resting-state was not significantly different (in any ROI) between FM and HC, we combined values from both cohorts when comparing task data to resting-state data. An illustrative example of power spectrums computed for a single WM ROI during the pain-stimulation task and resting-state is given in Fig. S4. For a few patients, the few seconds of the final pain block was not fully scanned. We therefore chose to omit the data obtained during the last block. The analysis of signal power at the task frequency is therefore based on data recorded during the first nine repetitions of the pain-stimulation block.

### Statistical analysis

Statistical significance (pain task > resting-state) was assessed using permutation tests (number of permutations = 10 000) and the threshold of significance was set to *q* < 0.01 using the false discovery rate method for correction for multiple comparisons. Since the time-locked activation analysis only considers the fundamental periodicity of BOLD signal change in WM and GM ROIs, we do not make any assumptions of the shape BOLD signal changes in response to pain stimulation and appraisal, respectively.

In an attempt to further exclude the possibility that residual subject head-motion related artefacts had a significant impact on our results, we performed an additional analysis of the power spectrum amplitude at the experimental task frequency for the BOLD signal extracted from regions within spaces filled with CSF. Regions encompassing CSF spaces were selected due to their high signal intensity on T_2_*-weighted MRI images and sensitivity to head-motion related signal changes. We used the CSF probability mask (‘csf.nii’, thresholded at 95% probability) supplied with the SPM software package. The power of the CSF signal at 0.026 Hz was computed in both cohorts. A two-sided *t*-test (permutation tests, *N* = 10 000) did not yield any significant difference at *P* < 0.05 (see Fig. S5).

## Results

### Pain ratings during the fMRI pain task

Patients rated their perceived pain intensity after each pressure stimulus (10 blocks) on a VAS (0–100 mm). A large majority (41 out of 54) of the HC patients reported low levels of perceived pain (mean < 20). In contrast, more than half (26 out of 41) of FM patients reported mean VAS scores > 40. The average VAS scores (across blocks) are given in Fig. S6.

### Time-locked BOLD activation in WM

**Figure 1:**
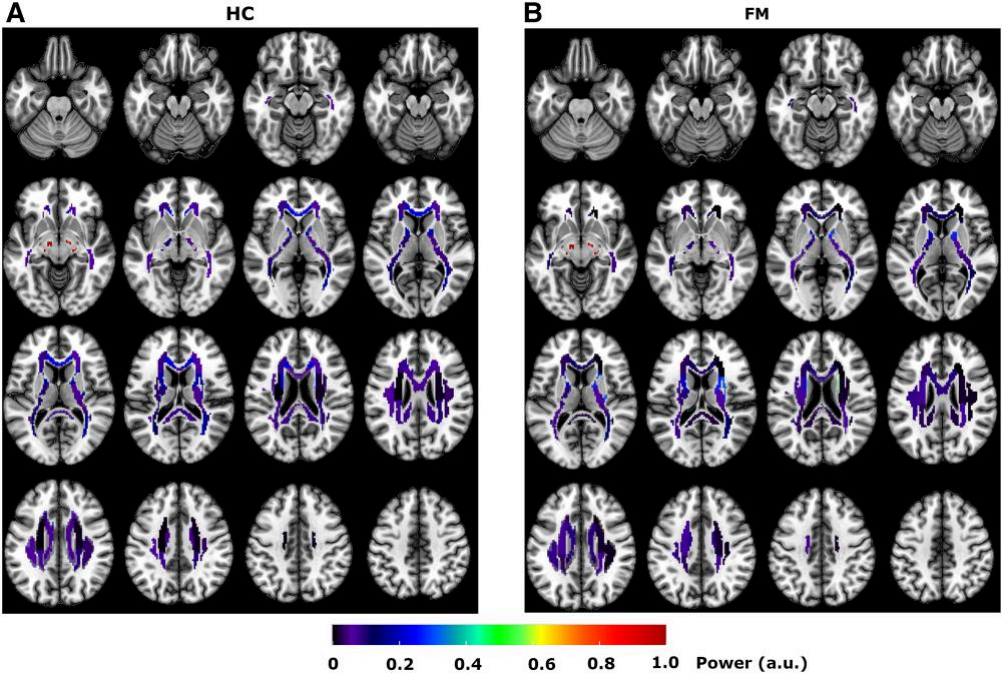
**Signal power in WM tracts.** Maps of the mean (across patients) and normalized signal power in WM tracts at the task frequency (0.026 Hz) for HC **(***N* = 54, **A**) and patients with FM (*N* = 41, **B**). WM tracts were parcellated into 49 regions using the International Consortium for Brain Mapping (ICBM) atlas.^26^ To minimize signal contributions from neighbouring GM voxels, only WM voxels that passed a 95% probability of residing in WM were considered in the analysis (see Methods section for further details). This requirement constrained our analysis of peak BOLD signal power to 29 out of the 49 WM ROIs defined in the ICBM atlas (see also Table S2).

**Figure 2:**
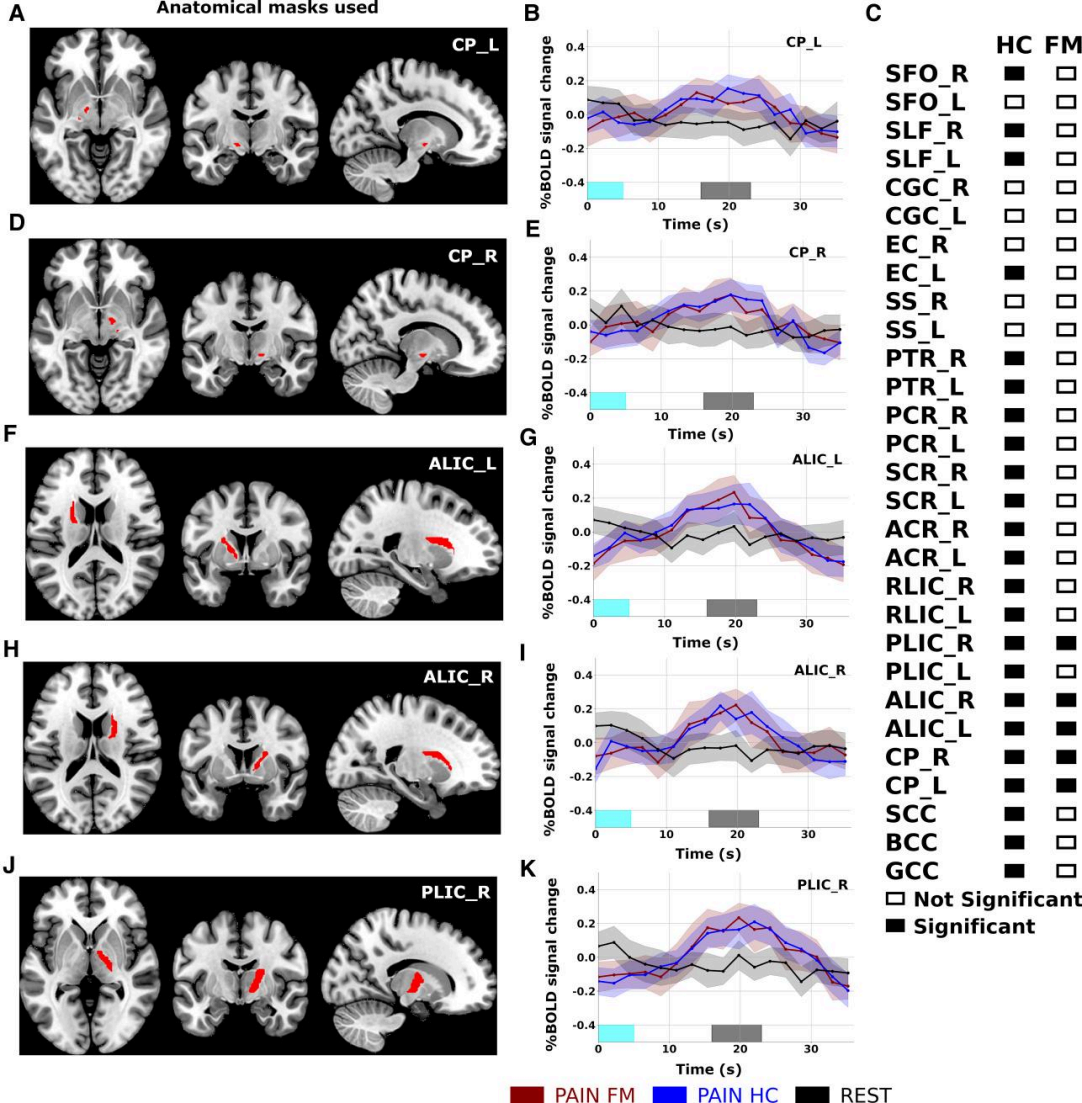
**Time-locked WM fMRI activations.** Five WM ROIs showed significantly larger WM BOLD signal power in both cohorts at task frequency (0.026 Hz) during the pain task compared to resting-state in FM patients (*N* = 41) and HCs (*N* = 54). The amplitude of BOLD power at task frequency was computed for the pain task and compared to the corresponding frequency bin of for resting-state within cohorts using permutation tests (number of permutations = 10 000, significance level *q* < 0.01, FDR corrected). Significant differences in WM BOLD signal power at the task frequency were present in the bilateral CP (**A** and **B**: left CP; **D** and **E**: right CP), bilateral ALIC (**F** and **G**: left ALIC; **H** and **I**: right ALIC), right PLIC (**J** and **K**). Panel **C** shows the results for all 29 WM ROIs. Time-locked brain activation profiles for the pain task (duration of applied pressure is marked in cyan, and appraisal of pain is marked in grey) were computed by first averaging across individual task repetitions (nine blocks of pain-stimulation and pain appraisal per task) and subsequently across patients in each cohort. Shaded areas in the time-locked BOLD signal plots mark the 95% confidence intervals. WM region abbreviations: SFO, superior fronto-occipital fasciculus; PCT, pontine crossing tract; SLF, superior longitudinal fasciculus; CGC, cingulum (gyrus); EC, external capsule; SS, sagittal striatum; PTR, posterior thalamic radiation; PCR, posterior corona radiata; SCR, superior corona radiata; ACR, anterior corona radiata; SCC, splenium of corpus callosum; BCC, body of corpus callosum; GCC, genu of corpus callosum; L, left; R, right. See also Table S2.

For WM ROIs, the average (normalized) peak power at task frequency (0.026 Hz) in the FM and HC cohorts are presented in Fig. 1, representing pain perception and appraisal during BOLD fMRI scanning. Overall, the spatial distribution of WM BOLD signal power was similar across cohorts, with a few differences. For example, the mean power in the genu of the corpus callosum (GCC) was larger in HC than FM during the pain task. Permutation tests of WM BOLD signal power at the task frequency relative to resting-state resulted in 23 (out of 29) WM ROIs significantly active in HC, while five regions in the FM cohort were significantly different from rest (Fig. 2C). A direct comparison of signal power between cohorts at the task frequency for the pain task (2-sided permutation test at *q* < 0.01, FDR corrected) did not reveal any significant differences in any WM ROI.

All five WM regions that were significantly active in the FM cohort were also significantly active in the HC. Details regarding their anatomical location and their time-locked WM BOLD activation profiles are given in Fig. 2. Significant time-locked WM BOLD activity that was shared across cohorts included the bilateral cerebral peduncle (CP), bilateral anterior limb of the internal capsule (ALIC) and the right posterior limb of the internal capsule (PLIC). Additionally, time-locked WM BOLD activity was significantly higher during the pain task compared to resting-state in HC for the majority of investigated WM ROIs (see also Fig. 2C). For completeness, a full account of WM ROI power spectrum (frequency range: 0–0.227 Hz), distribution of peak power at task frequency and time-locked BOLD activation time-course is given in Fig. S7.

### Time-locked BOLD activation in GM

**Figure 3:**
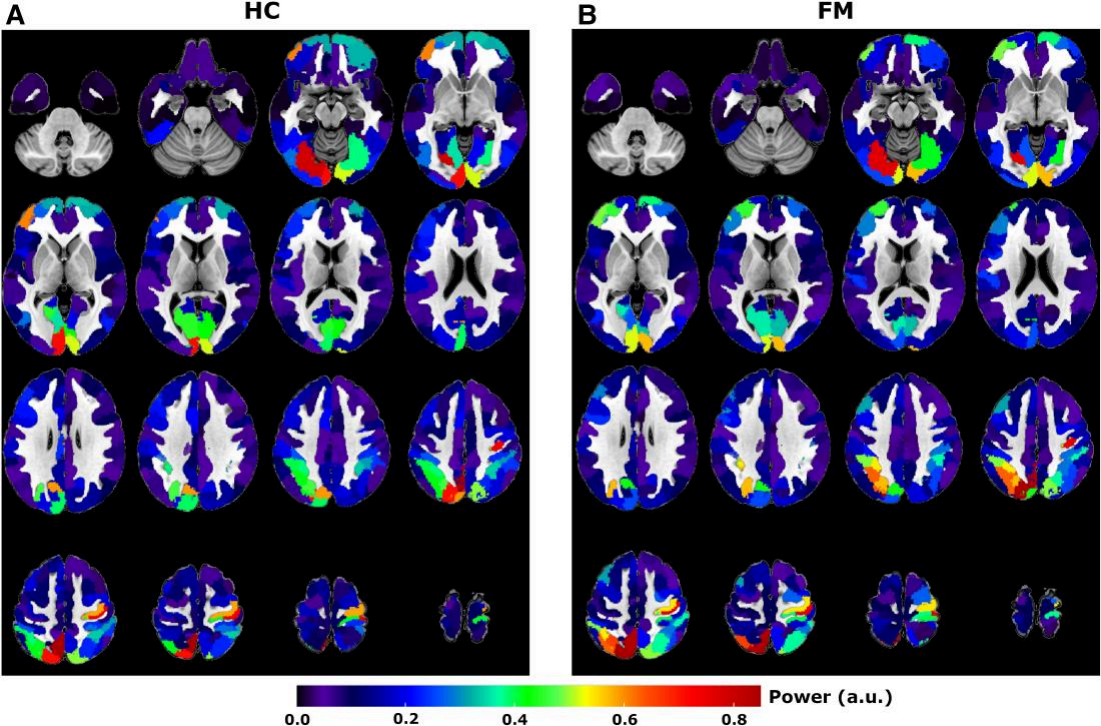
**Signal power in GM.** Maps of mean signal power in GM at the task frequency (0.026 Hz) for HC (*N* = 54, **A**) and FM cohort (*N* = 41, **B**). Average BOLD signal time-courses were extracted from 200 ROIs in the cerebral cortex using the Yeo–Schaefer atlas^27^ and their corresponding power spectrum were computed.

Next, we performed an analysis of time-locked BOLD activity in GM ROIs. The average peak power at the task frequency for all GM ROIs is presented in Fig. 3. We note the presence of relatively large amplitudes in task-related signal power for the visual, superior parietal, and the prefrontal cortex. The strongest signal power was found in the right somatomotor cortex, contralateral to the painful stimulation. Similar to WM ROIs, differences in mean BOLD signal power between FM and HC cohorts were detectable but relatively small (Fig. 3). Statistical significance testing of peak power at task frequency using permutation tests (relative to resting-state) showed that 168 out of 200 GM ROIs were significantly active in FM patients, whereas 169 out of 200 GM regions were significantly active in the HC (permutations tests, number of permutations = 10 000, *q* < 0.01, FDR corrected for multiple comparisons). A complete list of all GM ROIs is given in Table S3.

A direct comparison of the mean BOLD signal power during the pain task between FM and HC at task frequency yielded three GM ROIs located in the visual cortex, dorsal attention network and precuneus (all in the right hemisphere). Further details are provided in Fig. S8. It should be noted that for the visual cortex and the precuneus ROIs, the signal power at task frequency compared to resting-state was not significant in any cohort. In the case of the ROI in the dorsal attention network, the signal power at task frequency was not significantly different from resting-state in the FM cohort (see also Table S3). Given this context, we believe it is prudent to consider GM ROI signal power differences between cohorts cautiously.

**Figure 4:**
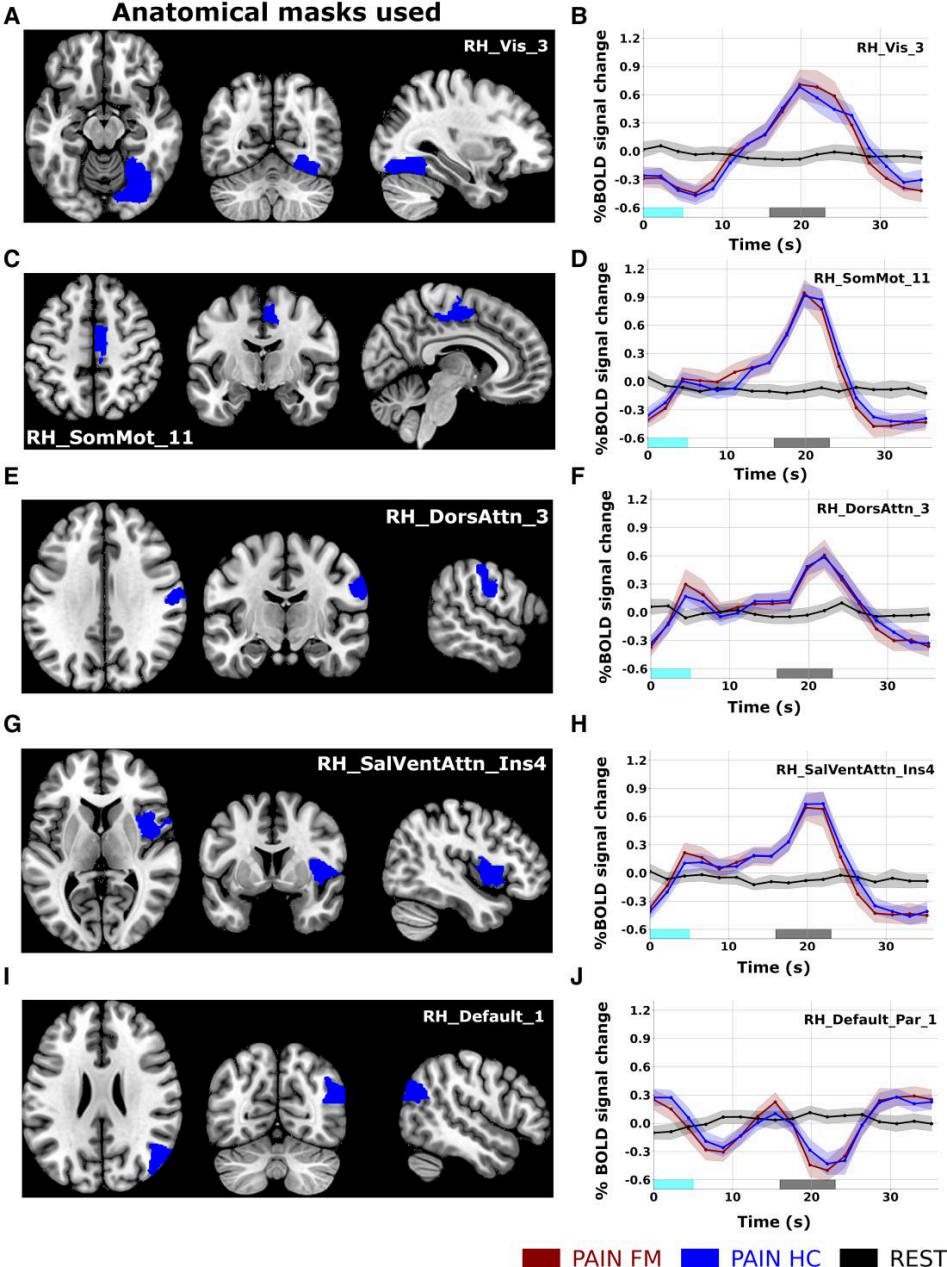
**Time-locked GM fMRI activations.** Selection of ROIs showing the variability of significantly active compared to resting-state time-locked GM BOLD activity profiles during the pain-stimulation task in FM patients (*N* = 41) and HCs (*N* = 54) (permutation tests, number of permutations **=** 10 000, significance level *q* < 0.01, FDR corrected). (**A** and **B**) Right visual cortex; (**C** and **D**) right foot/leg area in the somatomotor network; (**E** and **F**) right dorsal attention network; (**G** and **H**) right insula in the saliency/ventral attention network; and (**I** and **J**) right superior parietal lobule in the default mode network. The duration of applied pressure is marked in cyan, and the phase rating of perceived pain is marked in grey. Shaded areas in the time-locked BOLD signal plots mark the 95% confidence intervals (see also Fig. S8). GM region abbreviations: Vis, visual network; SM, somatomotor; SalVentAtt, ventral salience attention network; DorAtt, dorsal attention network; Default, default mode network; RH, right hemisphere; LH, left hemisphere.

There was a large variability of time-locked GM BOLD signal time-courses in response to the pain task. To exemplify this variability, the results from a subset of GM ROIs are shown in Fig. 4. For the case of the visual cortex (Fig. 4A and B), we observe a lack of an initial response to the pressure pain stimulus, but rather a gradual increase in the time-locked BOLD signal culminating during the appraisal phase. Conversely, the foot/leg area of the right somatomotor cortex (Fig. 4C and D) and the right dorsal attention network (Fig. 4E and F) showed an increased BOLD amplitude in response to the pain stimulation, followed by a subsequent peak during the appraisal period. A similar BOLD response profile was found in the right insular cortex (Fig. 4G and H). In contrast, for the right superior parietal lobule (a key region in the default mode network), we note a reversed BOLD signal profile with troughs accompanying both the pain stimuli as well as for the subsequent appraisal phase (Fig. 4I and J). A full account of all time-locked GM ROI signal time-courses is given in Fig. S9.

**Figure 5:**
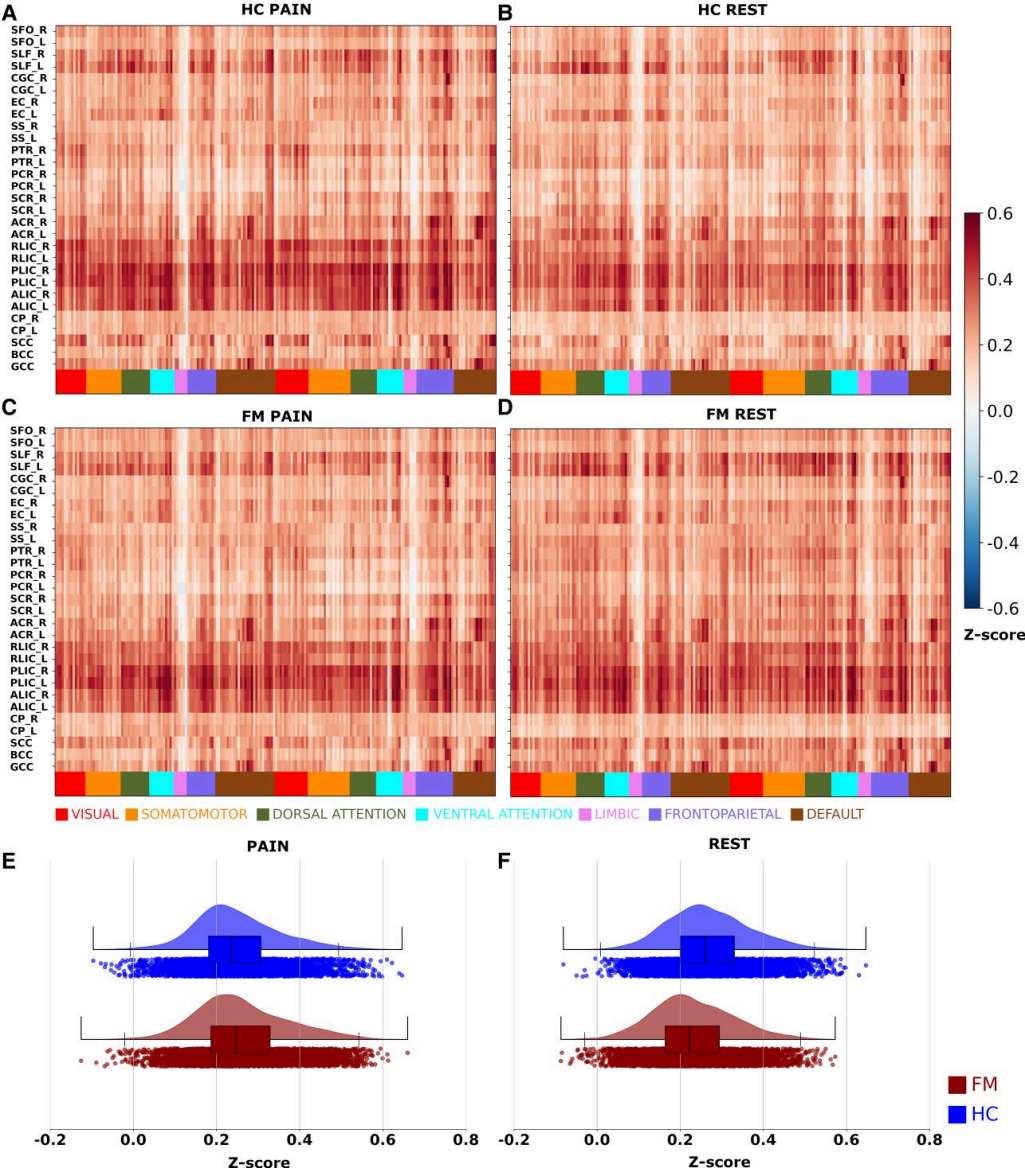
**Correlation maps for WM and GM region-of-interests in FM patients (*N* = 41) and HCs (*N* = 54).** Maps of the mean correlation values of BOLD signal time-courses extracted from WM ROIs (see also Figs 2 and 3) and GM ROIs (see also Fig. 4, Table S3 and Figs S8 and S9) during the pain-stimulation task (**A** and **B**) and resting state (**C** and **D**). Distributions of standardized correlation values for all patients (HC and FM cohorts) are shown in **E** (pain-stimulation task) and **F** (resting-state). Prior to the computation of correlation values, all BOLD signal time-courses were detrended and standardized followed by a Fischer *z*-transformation. Statistical significance tests were performed using permutation tests (number of permutations = 10 000, significance level *q* < 0.01, FDR corrected). WM region abbreviations: SFO, superior fronto-occipital fasciculus; PCT, pontine crossing tract; SLF, superior longitudinal fasciculus; CGC, cingulum (gyrus); EC, external capsule; SS, sagittal striatum; PTR, posterior thalamic radiation; PCR, posterior corona radiata; SCR, superior corona radiata; ACR, anterior corona radiata; SCC, splenium of corpus callosum; BCC, body of corpus callosum; GCC, genu of corpus callosum; L, left; R, right. See also Table S2.

To assess whether WM BOLD activity during pain stimulation is coupled to signal changes in neighbouring GM ROIs, we detrended and standardized BOLD signal time-courses (using the *nilearn.clean.signal* function in the nilearn python library) for all GM and WM ROIs (see also Figs 2 and 3), computed the correlation value between WM and GM ROIs and employed the Fischer *z*-transform. For comparison, we performed the identical procedure for resting-state runs. Maps and histograms for the standardized correlation values between BOLD WM and GM ROIs are shown in Fig. 5.

Overall, WM ROIs were positively correlated to cortical GM ROIs, while a few WM ROIs were negatively correlated with subcortical limbic regions. The overall pattern of positive correlations was preserved in both cohorts during the pain-stimulation task as well as during resting-state (Fig. 5A–D). In particular, we note the strong positive correlation between the five WM regions shown in Fig. 2, including the posterior and ALIC, and many cortical GM ROIs. A visual assessment of the histograms of correlation values shown in Fig. 5E and F suggests a slight shift towards more positive correlation values for the FM compared to the HC cohort during the pain task (Fig. 5E), whereas a shift towards higher correlation values for the HC cohort relative to the FM cohort is present during resting-state (Fig. 5F). A statistical test of the differences in mean correlation values revealed a small increase in the FM cohort compared to HC during the pain task (mean difference = 0.01, *P* = 0.00002, 2-sided permutation test, *n* = 10 000 permutations) whereas the opposite case was found during resting-state (mean differences = 0.04, *P* = 0.0002, 2-sided permutation test, *n* = 10 000 permutations). The dependence of task and cohort on the distribution of correlation values between WM and GM ROIs signal time-courses were found significant using the Kolmogorov-Smirnov test (pain-stimulation task: D = 0.054, *P* < 0.001; resting-state: D = 0.17, *P* < 0.001).

## Discussion

We applied a time-locked analysis of BOLD signal changes in WM and GM ROIs in response to a blocked and repetitive pain-stimulation task, and during resting-state fMRI in patients with chronic pain and HCs. To the best of our knowledge, this is the first study of time-locked WM BOLD signal changes during a pain-stimulation and appraisal task. As expected, the time-locked amplitudes of WM BOLD signal profiles were lower than for GM ROIs, typically by 50% or more. Yet, in both cohorts, we found significant time-locked WM BOLD activity during the pain task compared to resting-state in the CP and ALIC bilaterally, and in the right PLIC.

The CP is situated in the midbrain and is known to contain myelinated motor fibres of the corticospinal and corticobulbar tracts.^33^ The lateral parts of the CP contain fibre bundles that are destined for the contralateral lower extremities. The internal capsule is a deep subcortical structure that contains a concentration of afferent and efferent WM projection fibres. Anatomically, this is an important area because of the high concentration of both motor and sensory projection fibres. Afferent fibres pass from cell bodies of the thalamus to the cortex, and efferent fibres pass from cell bodies of the cortex to the CP of the midbrain. Fibres from the internal capsule contribute to the corona radiata. The ALIC is composed of thalamocortical fibres and frontopontine tracts. The thalamocortical tracts in the ALIC connect the anterior and medial thalamic nuclei with a large swath of the frontal lobe, including the orbitofrontal cortex, ventromedial prefrontal cortex, dorsal anterior cingulate cortex and the dorsal prefrontal cortex.^34^ The ALIC has been extensively studied in numerous psychiatric disorders, but also known to be active in cognitive processes such as ‘altering’—which has been described as the component of attention that is directed towards becoming and staying attentive towards one’s surroundings.^35^ The CP and the ALIC are anatomically closely related, because the internal capsule becomes the CP in the midbrain. The PLIC contains mainly corticospinal fibres that transmit sensory and motor neural signals between the body and the primary somatomotor cortex.

Thus, given the anatomical background for fibre tracts that have been found to be active in the current study, it seems plausible to suggest that a painful pressure applied to the left shin provokes a consistent, time-locked increase in BOLD signal activity in WM structures that are known to be closely associated with thalamocortical pathways that connect input originating from the peripheral nervous system with the cortical sensorimotor network. The involvement of the ALIC suggests that attentional monitoring plays an important part in the patterns of neuronal signalling between the cortex and the limbic system during the perception and appraisal of pain.

The thalamus plays a key role in pain processing^2^ and there are known functional and structural thalamic aberrations in chronic pain.^36^ A previous study from our group^37^ (change reference 13 to reference 37, see also AQ21) showed increased thalamus-prefrontal connectivity in response to cognitive behavioural therapy in FM, suggesting that restoring thalamocortical connectivity may normalize pain pathophysiology. Electrophysiological studies have revealed altered thalamocortical rhythms (‘thalamocortical dysrhythmia’) in patients with various forms of chronic pain, including neuropathic pain,^38^ complex regional pain syndrome^39^ and low-back pain.^40^ These disruptions impair signalling between the thalamus and cortex, affecting sensory-, motor-, and cognitive functions, yet the role of WM tracts in thalamocortical dysrhythmia has been elusive. Based on the findings in the present work, it is possible that thalamocortical disruptions in chronic pain are linked to altered WM signalling.

With regard to time-locked GM activity, many ROIs situated in the occipital, frontal, parietal and temporal cortices were found to be significantly active. The variability of time-locked GM BOLD signal profiles shown in Fig. 4 and Fig. S9 is noteworthy. For example, regions in the Saliency/Ventral Attention network (including the insula region) and somatomotor regions show a twin-peak-like response profile, including an initial peak in response to the pressure stimuli per se, followed by a second peak related to the appraisal phase (Fig. 4C and D, see also Fig. S9). In contrast, the visual, dorsal attention and control networks show mainly a single peak in BOLD activity related to the appraisal phase. We also note the marked inverted pattern of time-locked BOLD activation for the left superior parietal cortex (Fig. 4E), a region which is commonly ascribed to be a key node in the default mode network. The temporal diversity of time-locked BOLD signal activation profiles and their presence in large swaths of the cortex is in line with previous studies of whole-brain activation patterns even for simple sensory/motor task paradigms.^41^

Our finding of medium-to-large positive correlations between WM ROIs and many GM regions situated in cortical networks during the pain-stimulation task as well as during resting-state is noteworthy. In particular, the strong degree of correlation between WM tracts associated with the internal capsule (PLIC, ALIC, RLIC (Retrolenticular limb of the Internal Capsule), see also Fig. 3) and cortical areas during both resting-state and pain-stimulation stands out (Fig. 5A–D). Moreover, the overall distribution of correlation values is to some extent dependent on cohort and task with a slight increase in connectivity for the FM compared to the HC cohort during pain and vice versa during rest (Fig. 5E and F). Our finding of differences in the degree of mean WM-GM connectivity between cohorts is intriguing but given the small absolute change in correlation strength and the lack of anatomical specificity, we here refrain from speculation of potential mechanisms that might be involved.

While it has been shown that BOLD signal changes in WM are related to increases in neuronal activity due to external tasks^16,17,19,20^ or fluctuations in neuronal activity during resting-state conditions, the exact biophysiological origin is not fully understood. It remains to be determined whether WM BOLD signal changes are driven by increased metabolism in glia cells due to increased signal transduction or if they are coupled to a vascular change in the cortex.

### Limitations

The method of calculating BOLD signal power at a specific task frequency, here defined by the repetition of task blocks that incorporates both pain stimuli and appraisal of pain, combined with time-locked averaging of BOLD signal time-courses, implies that a-priori model of the BOLD HRF is not needed. Thus, this method provides a major advantage in that we are sensitive to any BOLD signal change, regardless of its shape, under the condition that it occurs repetitively across individual blocks in the task run. However, this also means that we cannot discuss BOLD responses due to the pain stimuli per se without also considering the signal changes due to the subsequent appraisal of the perceived pain. In other words, time-locked GM and WM ROI signal changes as presented in this study need to be discussed in the context of repetitive task epochs that contain both the perception of a painful pressure stimuli, but also the subsequent appraisal of painfulness. Hence, a block-design fMRI experiment without the pain appraisal phase would have been preferable, since it would allow for an unambiguous assessment of time-locked WM and GM BOLD responses to the perception of pain by and of itself. That being said, in this first study of pain-related WM BOLD signals changes, and its putative role in chronic pain, we opted to analyse fMRI data acquired during a task-based fMRI experimental design that also included a phase of appraisal of pain. The appraisal part is typically included in pain neuroimaging experimental designs that involve clinical cohorts so that differences in the degree of experienced pain across cohorts can be assessed. In future work, we intend to investigate the role of WM BOLD signalling in pain perception using task paradigms that are ideally suited for time-locked analysis of WM BOLD signal changes, e.g. by excluding the pain appraisal part.

Another possible concern is the presence of temporal jittering intervals in the pain task (i.e. the time-periods ISI1 and ISI2 as shown in Fig. S1). Jittering is typically introduced in fMRI task runs to mitigate unwanted predictability on behalf of the participant as well as to enforce an increased temporal variability of the sampling points of the BOLD HRF. In the case of a time-locked activation analysis, jittering is not desired since it could potentially lead to a broadening of the task-related peak in signal power in the frequency domain. However, as shown in Fig. S3, the variability in block length due to jittering was small compared to the total block length. This assessment is strengthened by the presence of sharp peaks at the task frequency in the power spectrums shown in Figs S4 and S7 (the peak in BOLD signal power typically reached its maximum at the frequency bin corresponding to 0.026 Hz). Hence, we believe that the temporal jittering included in the pain task paradigm employed had little impact on our time-locked analysis of BOLD signal changes in white and GM ROIs.

Although we found differences in time-locked activation profiles between cohorts when comparing the pain-stimulation task to resting-state, no significant differences in WM ROIs between cohorts were found for perceiving and appraising painful stimuli. The obvious caveat of inadequate sample sizes needs to be kept in mind when considering null results. In particular, given the overall smaller amplitude of WM compared to GM BOLD signals, this is a concerning issue. On the other hand, our sample of fMRI data acquired in 41 FM patients is from a clinical investigative standpoint not to be regarded as overly small. Rather, it is on par, or larger than, recently published functional neuroimaging studies of FM.^42-44^ Moreover, one also needs to keep in mind the fact that some of our FM patients scored low on the degree of pain perceived from the 300 kPa pressure stimuli (Fig. S6). Nevertheless, future studies on WM BOLD signal changes in FM likely would benefit from even larger sample sizes than used here. This is particularly important as the pathophysiology of FM is heterogenous with both peripheral and central nervous system aberrations as well as neuroimmune mechanisms implicated.^5^

## Conclusions

In summary, we have presented results that, for the first time, show that fibre tracts involved in thalamocortical pathways, which facilitate neuronal signalling between the peripheral nervous system and primary somatomotor cortical regions, show consistent time-locked BOLD activity in response to a task where patients perceive and appraise painful stimuli. It is noteworthy that changes in WM BOLD signal were substantially smaller in amplitude than for GM ROIs. Consistent patterns of time-locked WM BOLD activity were found in the bilateral ALIC and CP and the contralateral posterior limb of internal capsule. While no significant differences in pain-stimulation WM BOLD responses were found between cohorts, we observed a notable distinction when comparing pain-stimulation activity to resting-state. Specifically, time-locked BOLD activity in HC was significantly active in 23 out of 29 WM ROIs, whereas only 5 WM ROIs met the statistical threshold in the FM cohort. As mentioned earlier, it cannot be ruled out that the large difference between cohorts with regard to pain stimulus time-locked WM activity versus resting-state may be due to limited power to detect WM BOLD signal changes. Future studies using larger sample sizes and using tailored pain task fMRI experimental designs could help clarify this issue and provide deeper insights regarding the interplay between WM and GM BOLD signal changes related to the neuronal processing of pain in FM.

## Supplementary Material

fcaf409_Supplementary_Data

## Data Availability

Pseudonymized data can be shared with qualified academic investigators providing that: (i) the research collaboration complies with the ethical permission by the Ethical Review Board of Sweden; (ii) are in agreement with EU legislation on the general data protection and (iii) are regulated in a material transfer agreement.
