# Supplementary material for: White and gray brain matter activity during pain in fibromyaliga patients and healthy controls
Source: Brain Commun. 2025 Oct 17;7(6):fcaf409. doi: 10.1093/braincomms/fcaf409 (PMC12612582; doi:10.1093/braincomms/fcaf409)
Supplement: fcaf409_Supplementary_Data [file fcaf409_supplementary_data.docx]

SUPPLEMENTARY MATERIAL for Fransson et al., 2025

Inclusion Criteria:

- Female sex.
- age 20-65 years.

For the FM arm:

- Fulfilling the ACR 1990 fibromyalgia diagnostic criteria.
- Fulfilling the ACR 2016 fibromyalgia diagnostic criteria.

Exclusion Criteria:

- Autoimmune or inflammatory diseases (other than FM in the FM cohort).
- Other somatic diseases that could influence the study outcome (e.g. peripheral neuropathy etc.).
- Pain problems (other than FM in the FM cohort).
- Severe depression/anxiety that requires specific treatments.
- Medication with anticonvulsants, antidepressants or corticosteroids.
- Inability to refrain from NSAIDs, analgesics, sedatives or sleep medication 48 hours before examinations.
- Inability to communicate in Swedish or other factors that the investigator judges would interfere with the participation in the study.
- Smoking > 5 cigarettes/day.
- Pregnancy.
- Drug or alcohol abuse.
- Contraindications to skin biopsy (allergy to local anesthetics, hemophilia, medication with anticoagulants)
- Contraindications to fMRI (metal implants, pacemakers, or being left-handed (analysis difficulties)).

|  | Fibromyalgia | Healthy Controls | Group difference |
| --- | --- | --- | --- |
| Age (years) | 47.9 (10.2) | 50.7 (12.3) | P = 0.245 |
| Pain VAS_now | 51.9 (21.8) | 2.4 (5.4) | P < 0.001 |
| Pain VAS_average | 61.7 (17.4) | 5.4 (7.3) | P < 0.001 |
| Pain VAS_min | 27.0 (15.5) | 1.6 (5.1) | P < 0.001 |
| Pain VAS_max | 82.2 (12.0) | 14.4 (20.1) | P < 0.001 |
| WPI | 14.7 (3.3) | 1.4 (1.2) | P < 0.001 |
| SSS | 9.8 (1.6) | 2.7 (2.0) | P < 0.001 |
| FIQ | 59.1 (16.1) | NA | NA |
| PPT_arm (kPa) | 130.9 (60.1) | 257.1 (101.6) | P < 0.001 |
| P4_arm (kPa) | 159.9 (80.2) | 312.2 (101.2) | P < 0.001 |
| P7_arm (kPa) | 237.9 (113.2) | 456.4 (150.6) | P < 0.001 |
| PPT_leg (kPa) | 174.2 (68.8) | 311.6 (118.6) | P < 0.001 |
| P4_leg (kPa) | 237.3 (88.5) | 397.5 (142.8) | P < 0.001 |
| P7_leg (kPa) | 327.8 (132.7) | 532.9 (181.1) | P < 0.001 |

***Supplementary Table 1.*** *Subject characteristics. Mean values and standard deviations (SD) are reported. Pain VAS = visual analogue scale ratings of pain intensity, at time of examination (now), average during the past week, and min = minimal pain intensity and max = maximal pain intensity during the past week, 0 = “no pain” and 100 = “worst imaginable pain”. WPI = widespread pain index corresponds to number of painful body regions (0-19), SSS symptom severity score (0-12) (WPI and SSS form part of the 2016 Diagnostic criteria for fibromyalgia). FIQ = fibromyalgia impact questionnaire (0 = no impact and 100 = maximal impact). PPT = pressure pain thresholds, P4 = pressure corresponding to a pain rating of 4/10 (moderate pain), P7 = pressure corresponding to a pain rating of 7/10 (very strong pain).*

| **Nr** | **Abb.** | **Name** | **Vx** | **SPM>0.95** |
| --- | --- | --- | --- | --- |
| 1 | MCP | Middle cerebellar peduncle | 1848 | 0 |
| 2 | PCT | Pontine crossing tract (part of MCP) | 183 | 0 |
| 3 | GCC | Genu of Corpus Callosum | 1131 | 618 |
| 4 | BCC | Body of Corpus Callosum | 1727 | 959 |
| 5 | SCC | Splenium of Corpus Callosum | 1543 | 670 |
| 6 | FX | Fornix | 81 | 0 |
| 7 | CST_L | Corticospinal tract Left | 176 | 0 |
| 8 | CST_R | Corticospinal tract Right | 178 | 0 |
| 9 | ML_L | Medial lemniscus Left | 86 | 0 |
| 10 | ML_R | Medial lemniscus Right | 83 | 0 |
| 11 | ICP_L | Inferior Cerebellar Peduncle Left | 113 | 0 |
| 12 | ICP_R | Inferior Cerebellar Peduncle Right | 119 | 0 |
| 13 | SCP_L | Superior Cerebellar Peduncle Left | 123 | 0 |
| 14 | SCP_R | Superior Cerebellar Peduncle Right | 121 | 0 |
| 15 | CP_L | Cerebral Peduncle Left | 268 | 22 |
| 16 | CP_R | Cerebral Peduncle Right | 263 | 25 |
| 17 | ALIC_L | Ant. Limb of Internal Capsule Left | 407 | 175 |
| 18 | ALIC_R | Ant. Limb of Internal Capsule Right | 392 | 169 |
| 19 | PLIC_L | Pos. Limb of Internal Capsule Left | 501 | 418 |
| 20 | PLIC_R | Pos. Limb of Internal Capsule Right | 477 | 330 |
| 21 | RLIC_L | Retrolenticular limb of Int. Cap. Left | 316 | 294 |
| 22 | RLIC_R | Retrolenticular limb of Int. Cap. Right | 311 | 218 |
| 23 | ACR_L | Anterior corona radiata Left | 856 | 735 |
| 24 | ACR_R | Anterior corona radiata Right | 865 | 752 |
| 25 | SCR_L | Superior corona radiata Left | 920 | 896 |
| 26 | SCR_R | Superior corona radiata Right | 924 | 894 |
| 27 | PCR_L | Posterior corona radiata Left | 452 | 393 |
| 28 | PCR_R | Posterior corona radiata Right | 446 | 411 |
| 29 | PTR_L | Posterior thalamic radiation (OR) Left | 487 | 361 |
| 30 | PTR_R | Posterior thalamic radiation (OR) Right | 478 | 282 |
| 31 | SS_L | Sagittal striatum Left | 286 | 164 |
| 32 | SS_R | Sagittal striatum Right | 288 | 105 |
| 33 | EC_L | External capsule Left | 466 | 107 |
| 34 | EC_R | External capsule Right | 455 | 93 |
| 35 | CGC_L | Cingulum (gyrus) Left | 294 | 18 |
| 36 | CGC_R | Cingulum (gyrus) Right | 337 | 36 |
| 37 | CGH_L | Cingulum (hippocampal) Left | 153 | 0 |
| 38 | CGH_R | Cingulum (hippocampal) Right | 131 | 0 |
| 39 | SLF_L | Superior Longitudinal Fasciculus Left | 825 | 639 |
| 40 | SLF_R | Superior Longitudinal Fasciculus Right | 815 | 591 |
| 41 | SFO_L | Sup. Fronto-occipital Fasciculus Left | 59 | 32 |
| 42 | SFO_R | Sup. Fronto-occipital Fasciculus Right | 55 | 51 |
| 43 | IFO_L | Inf. Fronto-occipital Fasciculus Left | 263 | 1 |
| 44 | IFO_R | Inf. Fronto-occipital Fasciculus Right | 242 | 0 |
| 45 | UNC_L | Uncinate Fasciculus Left | 47 | 0 |
| 46 | UNC_L | Uncinate Fasciculus Right | 49 | 0 |
| 47 | TAP_L | Tapetum Left | 78 | 9 |
| 48 | TAP_R | Tapetum Right | 71 | 0 |

***Supplementary Table 2****. Spatial regions of interest (ROIs) in white brain matter as defined by the International Brain Consortium Mapping atlas. See also Figures 1 and 2. The number of voxels contained in each ROI is stated together with the remaining number of voxels after using the probabilistic white matter mask provided in the Statistical Parametrical Mapping (SPM) package (“white.nii”, thresholded at 95% probability that a given voxel is residing in white matter).*

*
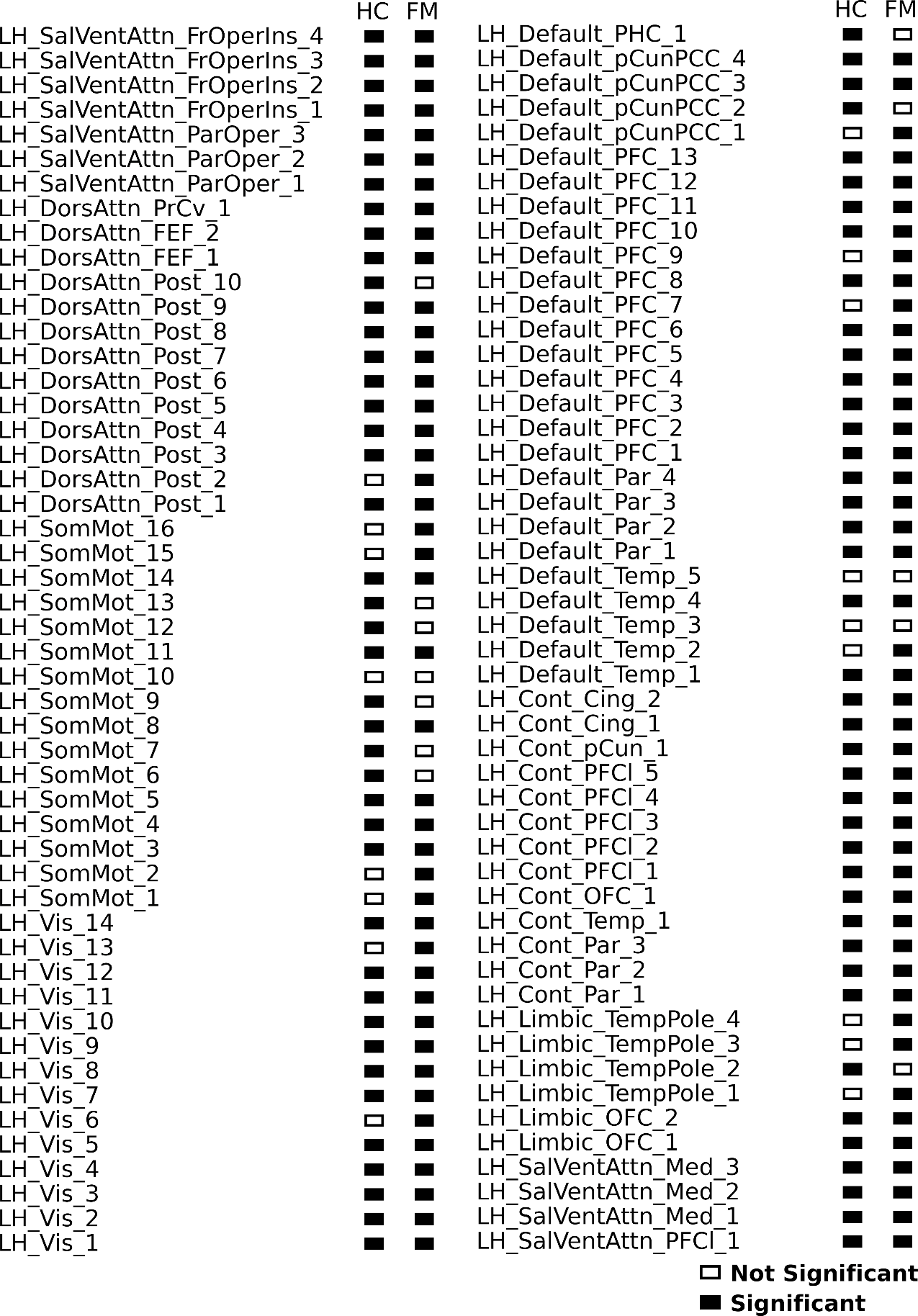
*

***Supplementary Table 3****. Significant differences in BOLD peak power (at task frequency 0.026 Hz) between the pain stimulation task and resting-state for all GM ROIs. Statistical significance was assessed using permutation tests (number of permutations = 10000) using the False Discovery Rate threshold (FDR) at q < 0.01 to correct for multiple comparisons. Filled square = Significant. Unfilled square = Not Significant. LH = Left Hemisphere. RH = Right hemisphere, Default – default mode network, DorsAttn – Dorsal Attention network, SalVentAttn – Ventral Saliency Attention network, Vis – Visual network, SomMot – Somatomotor network, Cont – Control network. Table is continued below.*

***
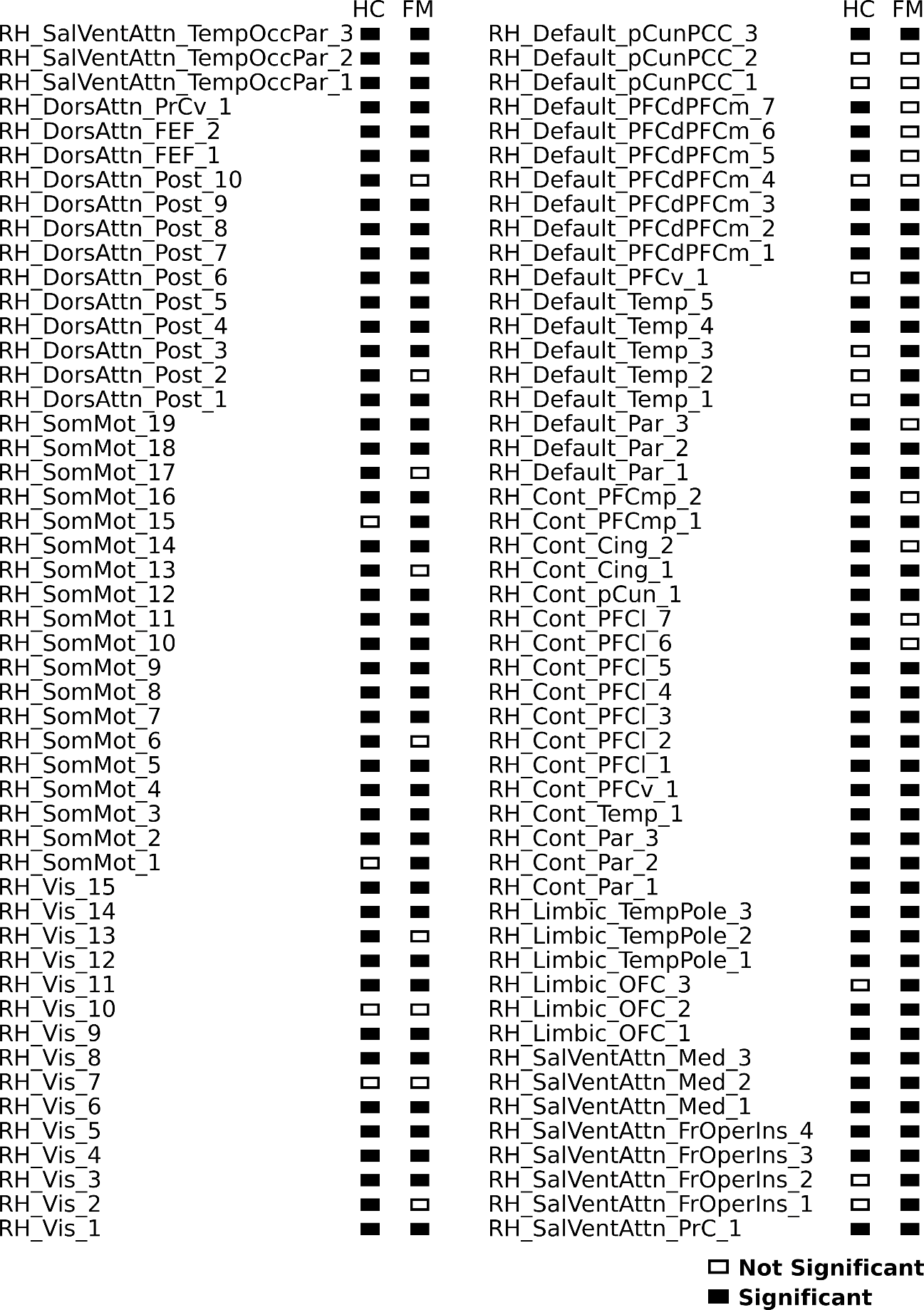
***

***Supplementary Table 3 cont.*** *RH = Right Hemisphere.*

SUPPLEMENTARY FIGURES


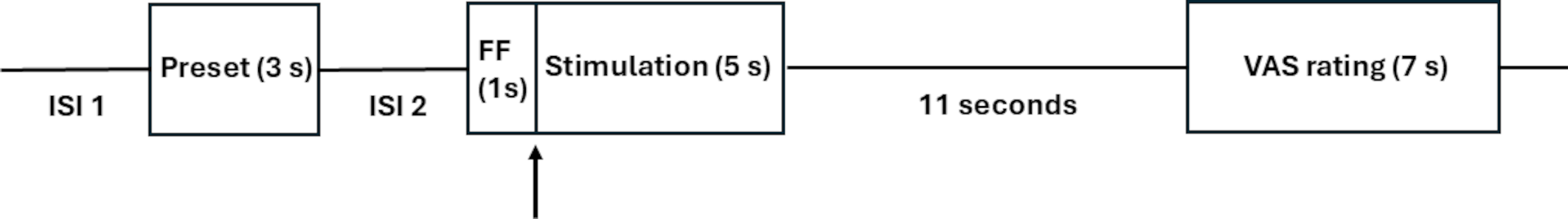


***Supplementary Figure 1****. Schematic timeline of the repetitive, block-based pain stimulation task. Each task block starts with a rest period (ISI1, Inter-Stimulus-Interval - a jittered time-interval between 1.5 and 3.5 seconds). Next, a preset phase is presented (when the automated pressure device descended the probe to a fixed position 5 mm above the skin of the left shin, followed by a second jittered rest period (ISI2, same jittering scheme as for ISI1). The second inter-stimulus-interval is followed by a brief (1 second) forewarning (FF, which included a flicker in the center of cross before a 300kPA pressure to the participant’s left shin is applied for 5 seconds. The pressure phase is followed by an 11-second rest period. The last phase of the task block is a 7 second visual presentation of a visual analog scale (VAS) rating of the preceding pain stimuli.*

*
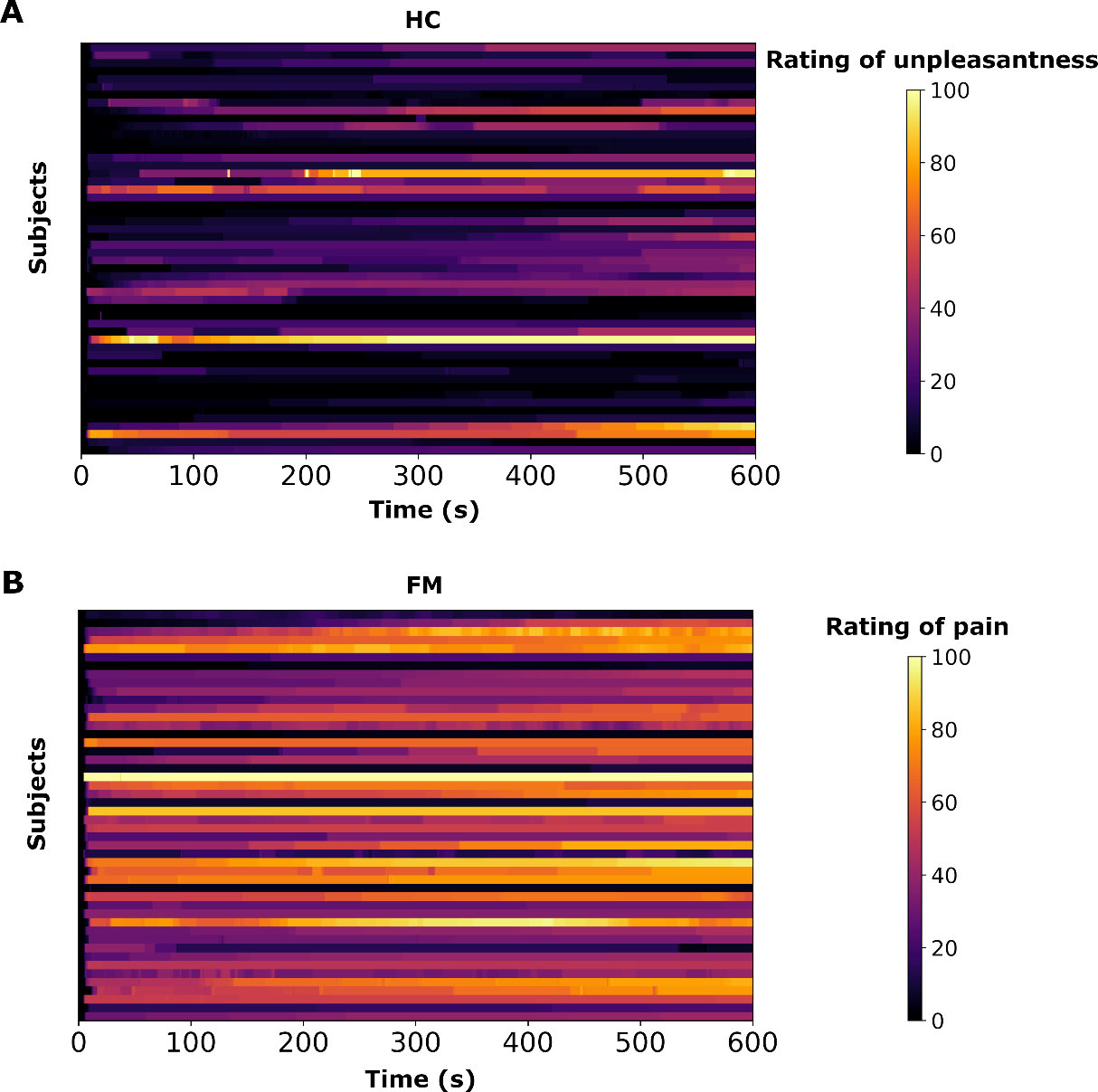
*

***Supplementary Figure 2****. Ratings of pain (FM, N=41) or physical unpleasantness (HC, N=54) during the 10-minute resting-state fMRI run. Participants continuously rated their perceived level of pain/physical unpleasantness on a visual analog scale (0-100 mm, VAS) throughout the resting-state run using a trackball device.*


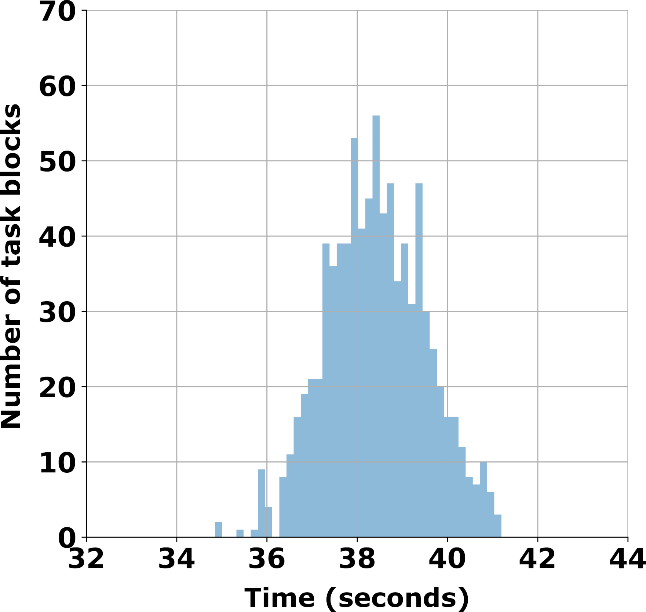


***Supplementary Figure 3****. Temporal spread of task-block lengths (collapsed over HC (N=54) and FM cohorts, N=41) due to the presence of temporal jittering (ISI1 and ISI2, see also Supplementary Figure S1) in the pain stimulation task. The mean task-block length (averaged across all blocks and participants) was 38.43 seconds (SD = 1.10 seconds), which resulted in an average task-block frequency of 1 / 38.43 = 0.026 Hz.*

*
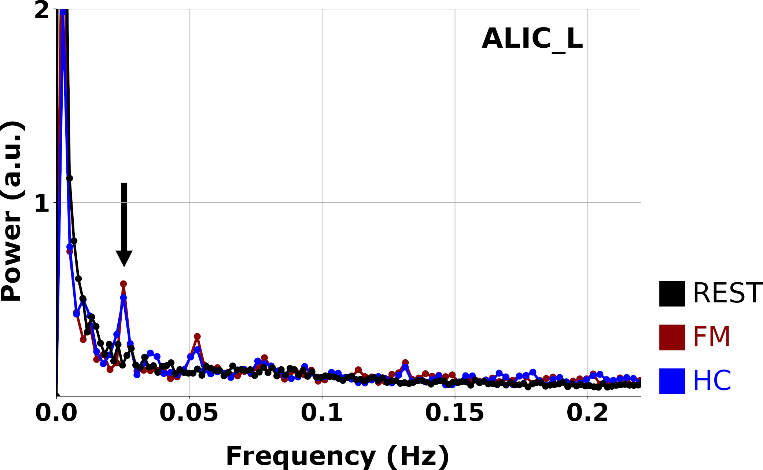
*

***Supplementary Figure 4****. An illustrative example of a BOLD signal power spectrum computed using the periodogram method (see methods section). The graph shows the power spectrum for the left anterior limb of the internal capsule (ALIC L) computed for the pain stimulation task and resting-state in both cohorts (FM: N= 41, HC: N = 54). The black arrow marks the frequency bin corresponding to the task frequency of 0.026 Hz.*

***
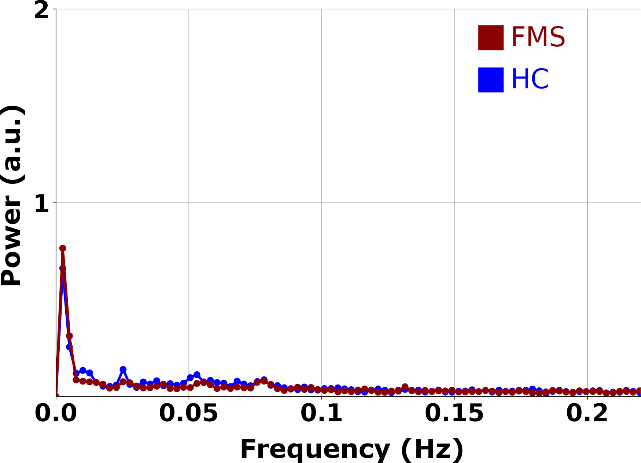
***

***Supplementary Figure 5****.* BOLD power spectrums for cerebrospinal fluid (CSF) regions in the brain for the control cohort (HC, N=54) and fibromyalgia participants (FM, N=41) for the pain task. There was no significant difference in mean CSF power between cohorts at the experimental frequency (0.026 Hz) (q>0.01, permutation test, number of permutations = 10000, 2-sided test, FDR corrected).

*
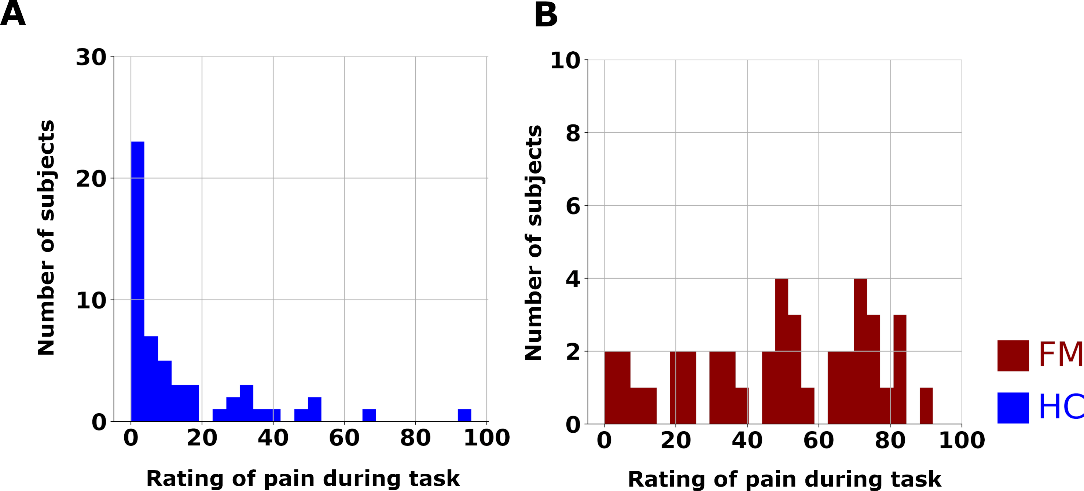
*

***Supplementary Figure 6.*** Ratings of pain during the fMRI pain stimulation task*.* Participants rated their degree of perceived pain after each pain stimuli (10 repetitions) on a visual analog scale (VAS, 0-100). The average VAS scores (across repetitions) are shown (mean FM = 48.5, mean HC = 14.2). FM: N=41, HC: N = 54.

*
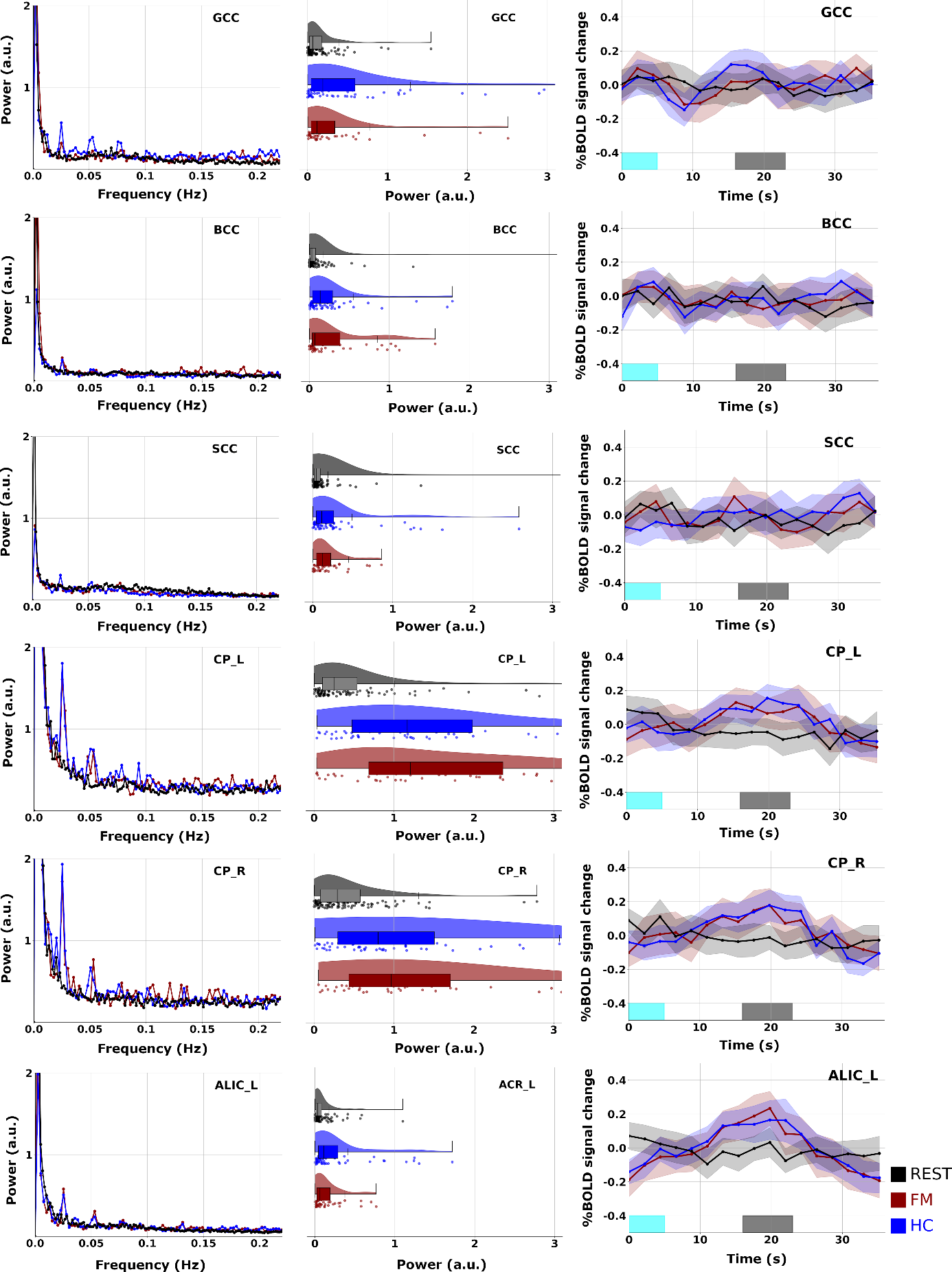
*

***Supplementary Figure 7****. BOLD power spectrums for all 29 white matter regions computed for the pain stimulation task, FM: N = 41, HC: N = 54. See also Figures 1 and 2 in the main text. Boxplots show the summary statistics, which include the minimum, first quartile, median, third quartile and maximum value of the peak BOLD power amplitudes at the task frequency (0.026 Hz). Time-locked brain activation profiles for the pain stimulation task were computed by averaging across blocks (9 task-blocks of pain stimulation and pain appraisal per fMRI run) and across participants in each cohort. The duration of applied pressure is marked in cyan and rating phase of perceived pain is marked in gray. Shaded areas in the time-locked BOLD signal plots mark the 95 per cent confidence intervals. White matter region abbreviations: SFO – Superior fronto-occipital fasciculus, PCT – Pontine crossing tract, SLF – Superior longitudinal fasciculus, CGC – Cingulum (gyrus), EC – External capsule, SS – Sagittal striatum, PTR – Posterior thalamic radiation, PCR – Posterior corona radiata, SCR – Superior corona radiata, ACR – Anterior corona radiata, SCC – Splenium of corpus callosum , BCC – Body of corpus callosum, GCC – Genu of corpus callosum, L – Left, R – Right. See also Supplementary Table 2. Statistical tests:* permutation tests, number *of permutations = 10000, q < 0.01, 2-sided test, FDR corrected). Figure continued below.*

*
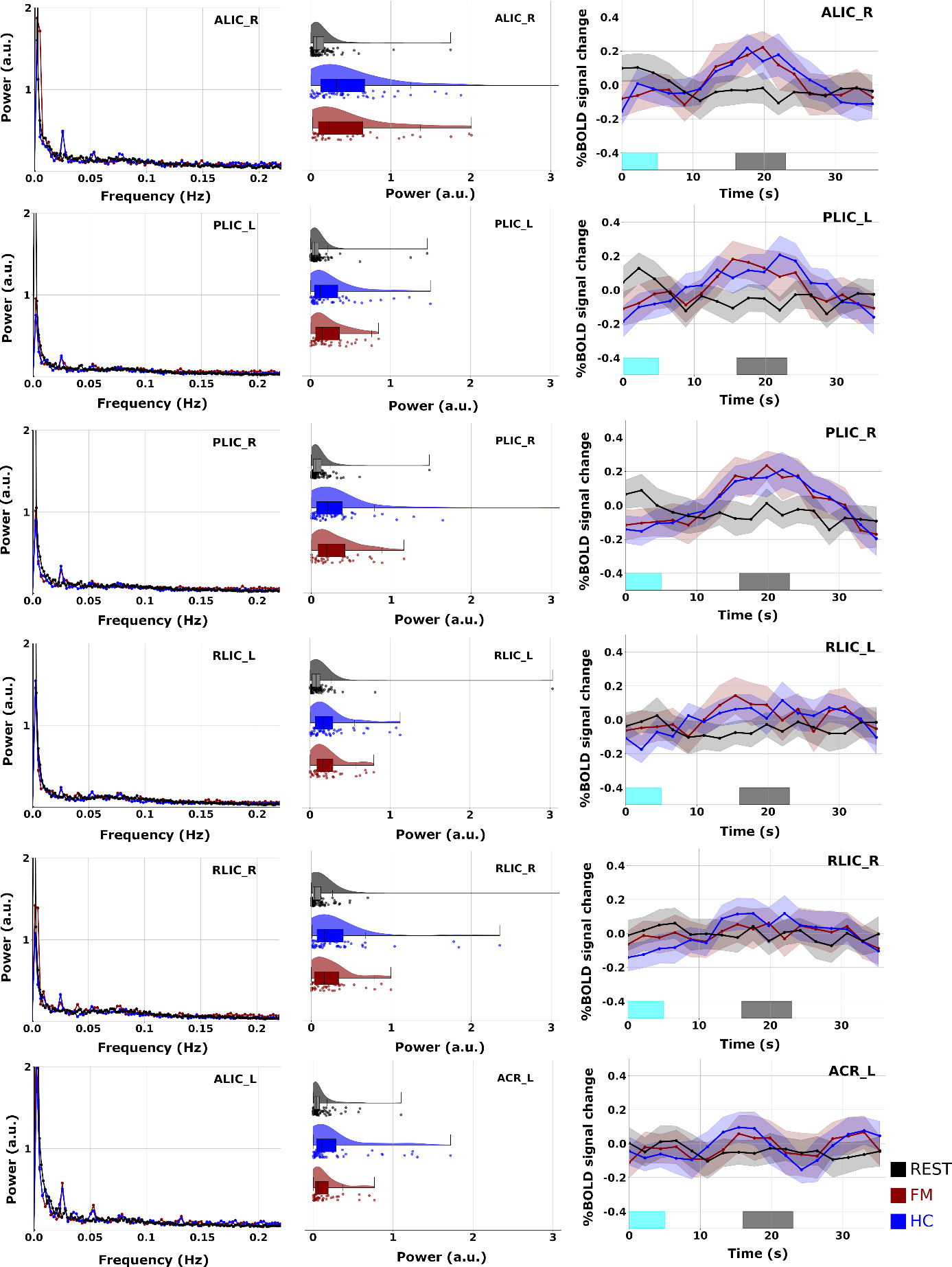
*

***Supplementary Figure 7****. Cont.*

*
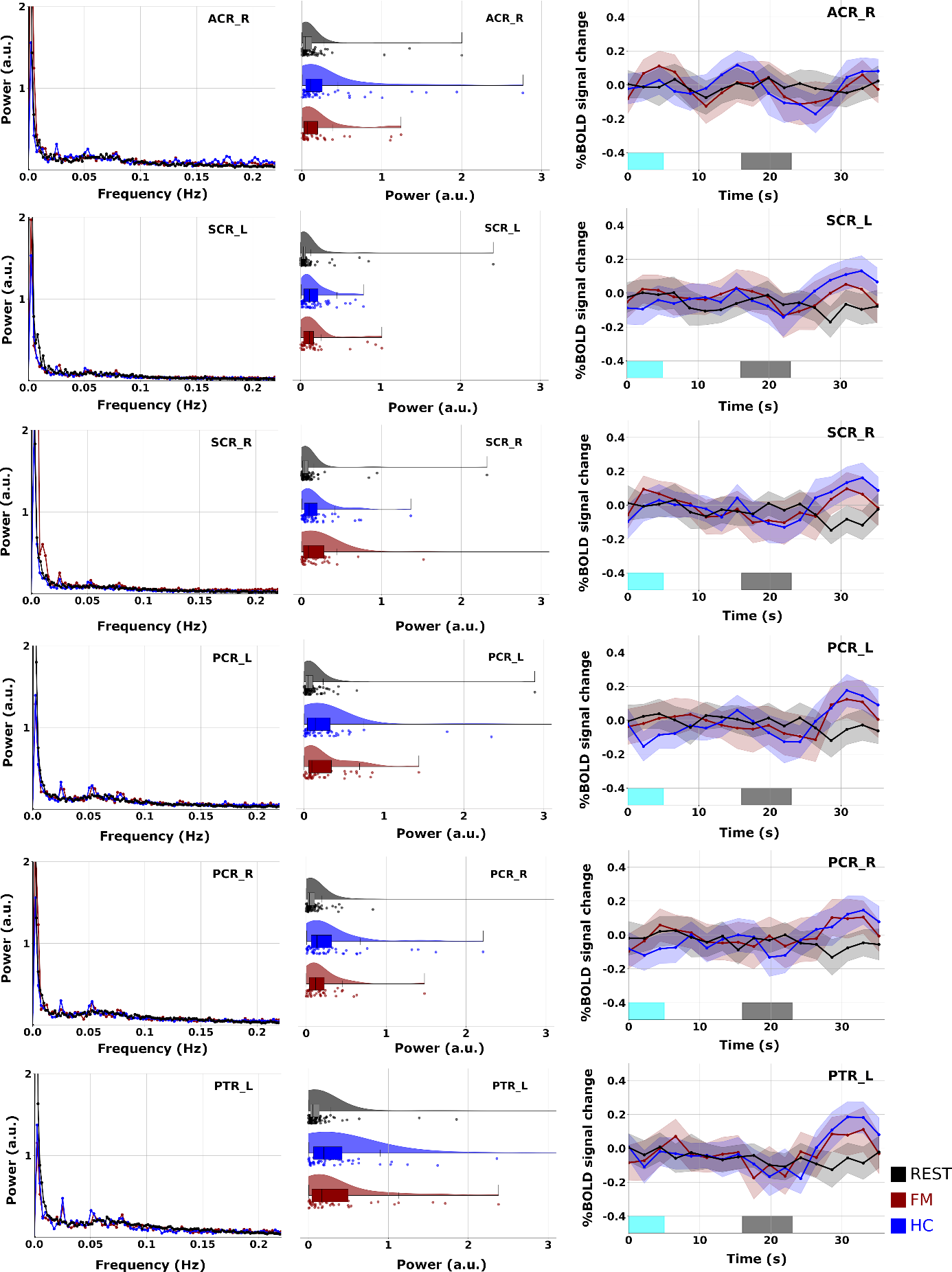
*

***Supplementary Figure 7.*** *Cont.*

*
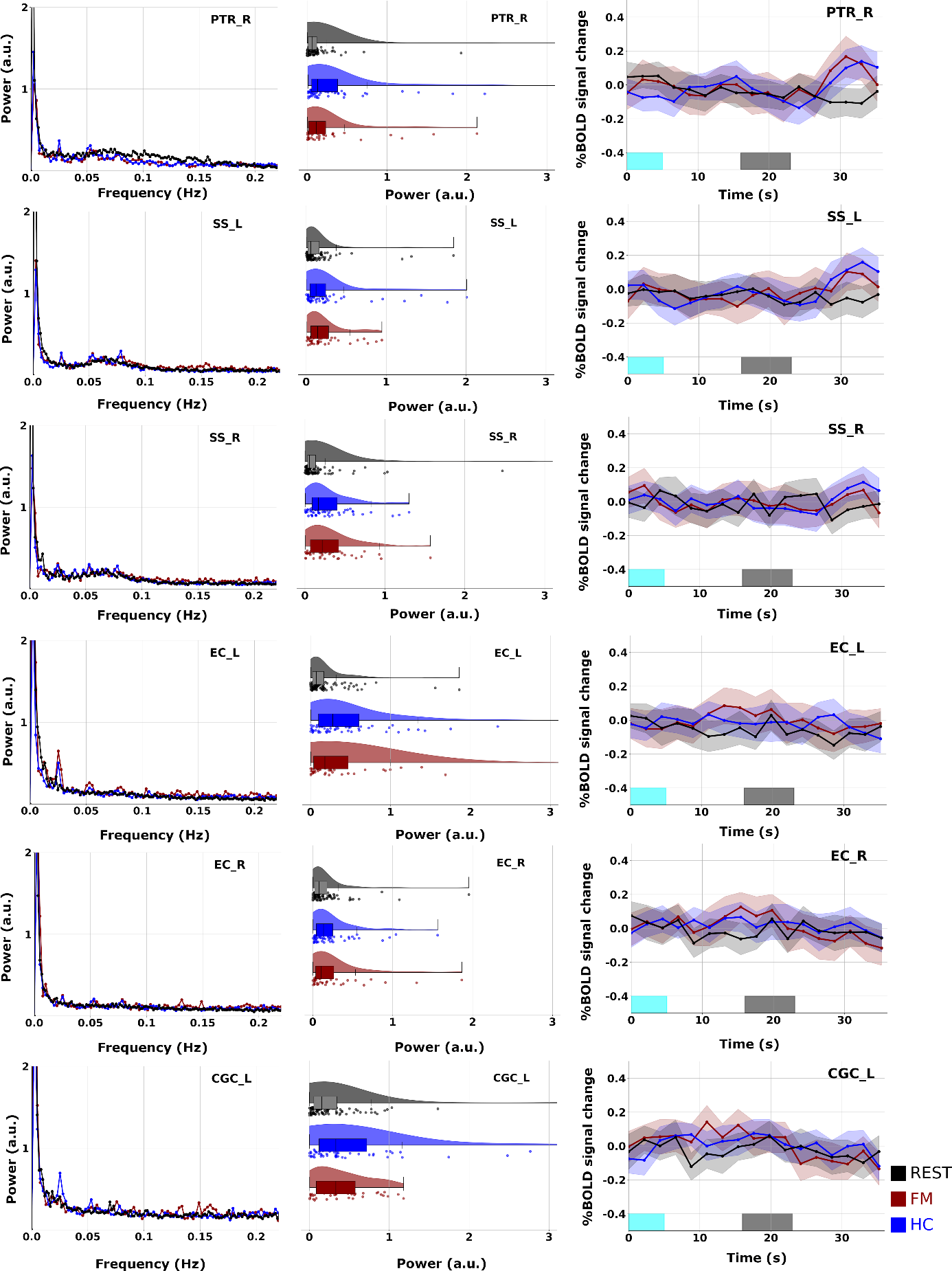
*

***Supplementary Figure 7.*** *Cont.*

***
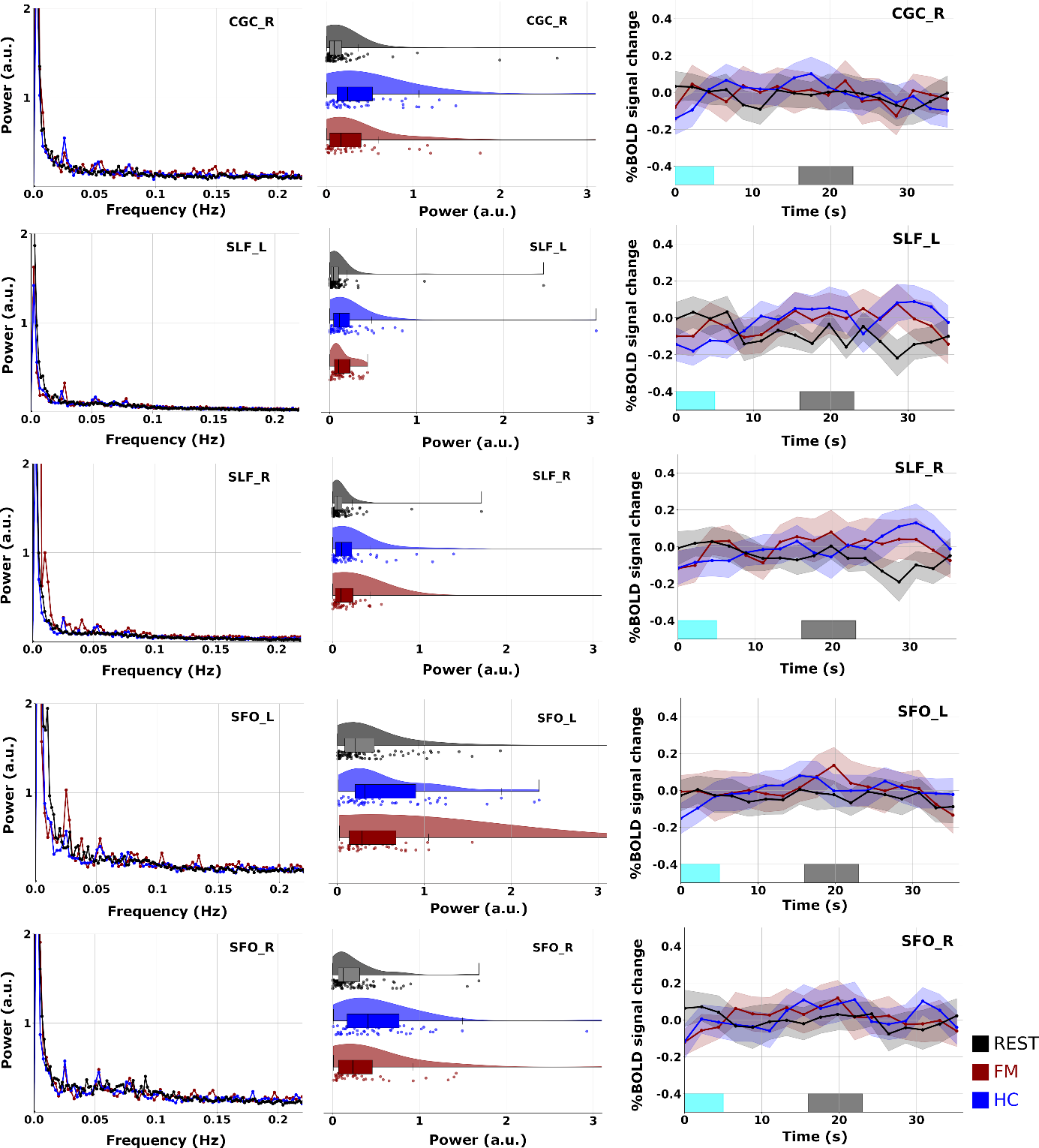
***

***Supplementary Figure 7.*** *Cont.*

***
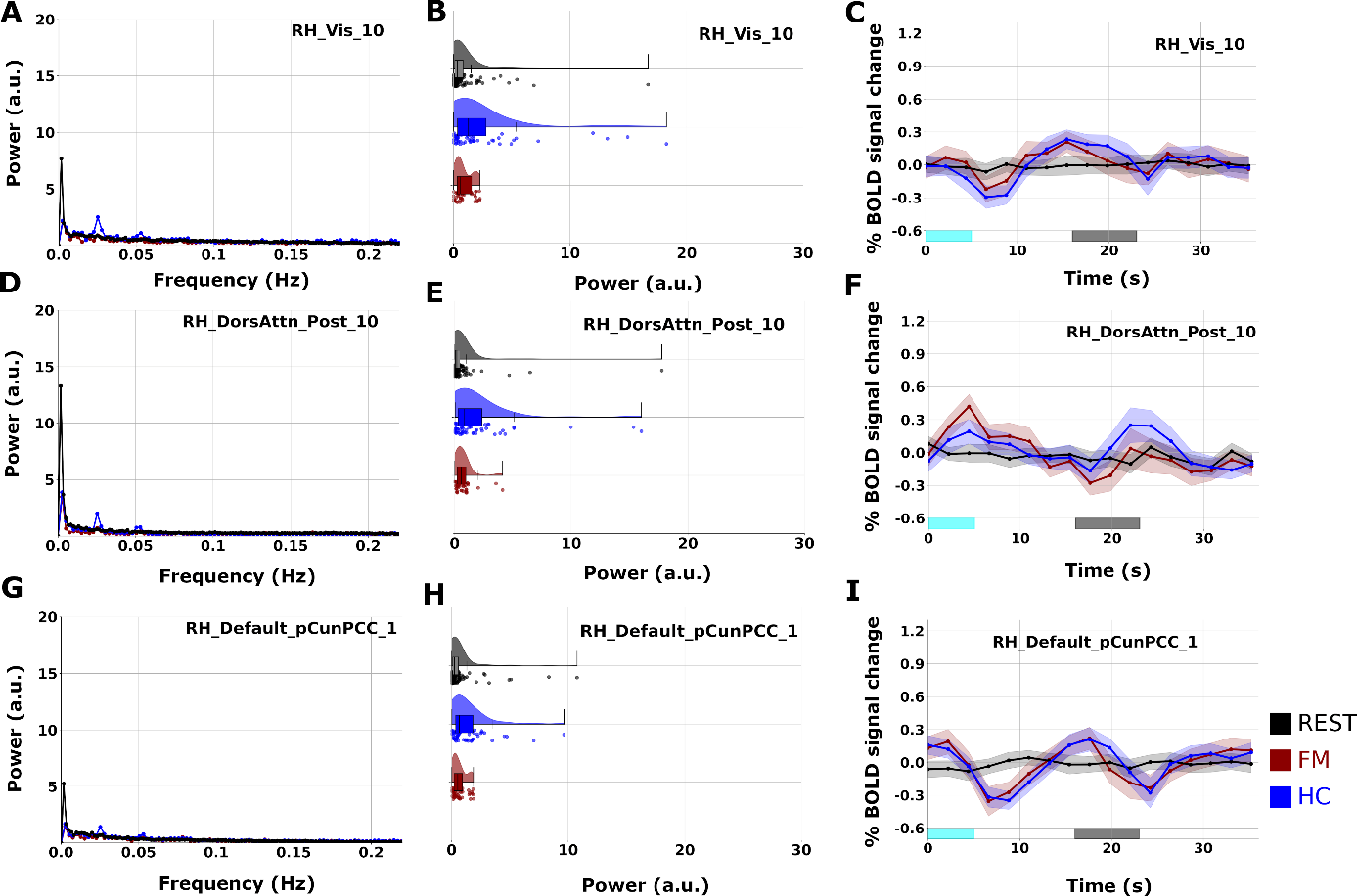
***

***Supplementary Figure 8.*** *Gray matter ROIs that showed a significant difference in peak power at the task frequency between cohorts (FM: N = 41, HC: N = 54, permutation tests, 2-sided, number of permutations = 10000, q<0.01, FDR corrected). See also Figures 3 and 4 in the main text. Note that for the ROIs in the visual cortex and the precuneus (panels A and C), the signal power during pain task compared to resting-state was not significant in any cohort. In the case of the ROI in the dorsal attention network (panel B), the signal power during the pain task was not significantly different from resting-state in the FMS cohort (see also Supplementary Table 3). RH = Right hemisphere, Vis = Visual network, DorsAttn = Dorsal Attention Network, Default = Default Mode Network. pCunPCC = precuneus posterior cingulate cortex, Post = Posterior.*

*
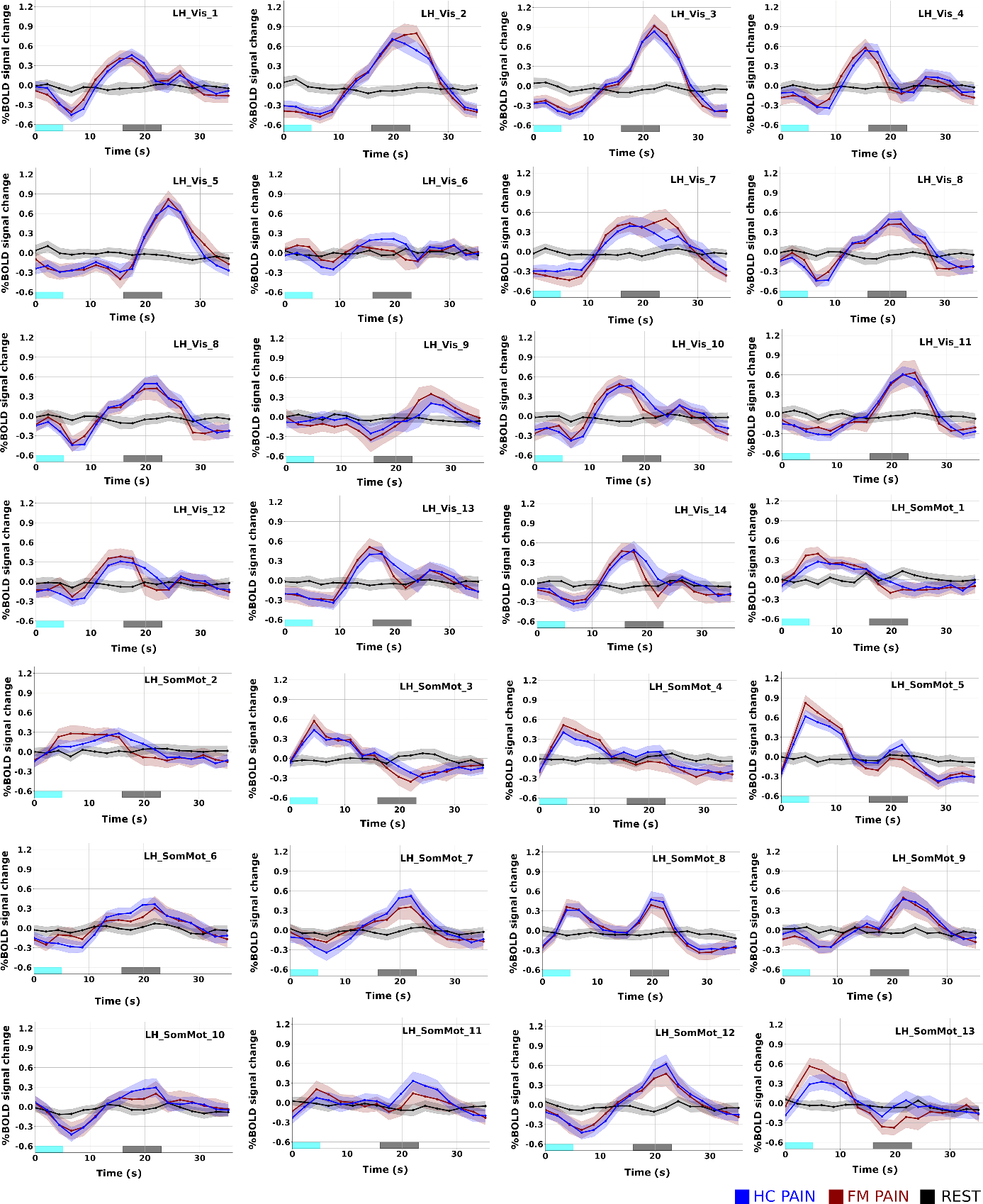
*

***Supplementary Figure 9.*** *Time-locked GM BOLD signal activation profiles (red = FM, N = 41, blue = HC, N = 54, black = REST (combined data from both cohorts)) for ROIs in the left hemisphere for the pain stimulation task. See also Figures 3 and 4 in the main text. The duration of the pain stimuli is marked in cyan, and duration of VAS rating period is marked in gray. Statistical tests: permutation tests, 2-sided, number of permutations = 10000, q<0.01, FDR corrected. SomMot = Somatomotor network, SalVentAttn = Ventral Saliency Attention network, Vis = Visual network, DorsAttn = Dorsal Attention network, Cont = Control network, Default = Default mode network. LR = Left Hemisphere, RH = Riggh Hemisphere. The figure is continued below.*

*
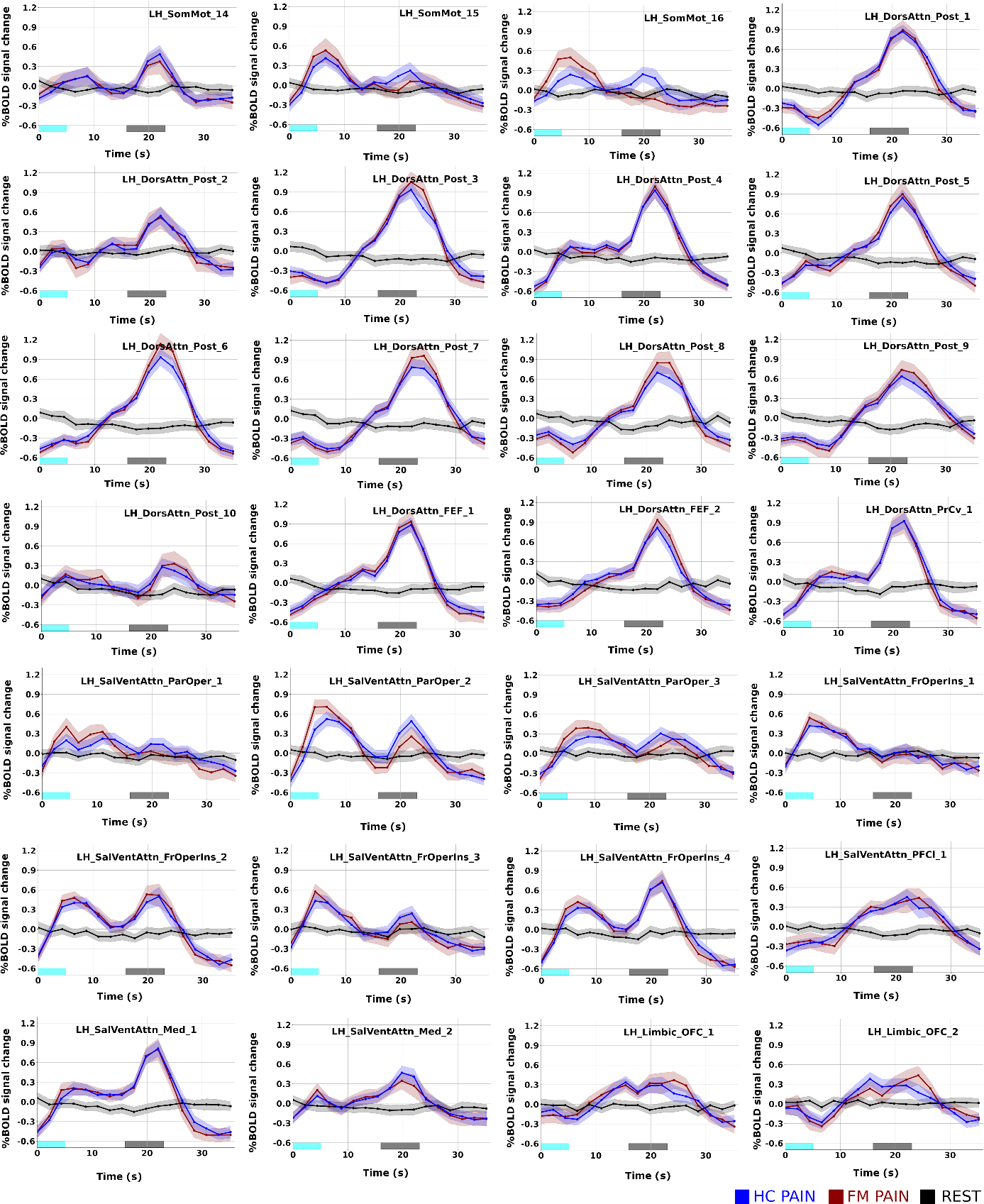
*

***Supplementary Figure 9 Cont.*** *Time-locked BOLD signal activation profiles for GM in the left hemisphere (LH).*

*
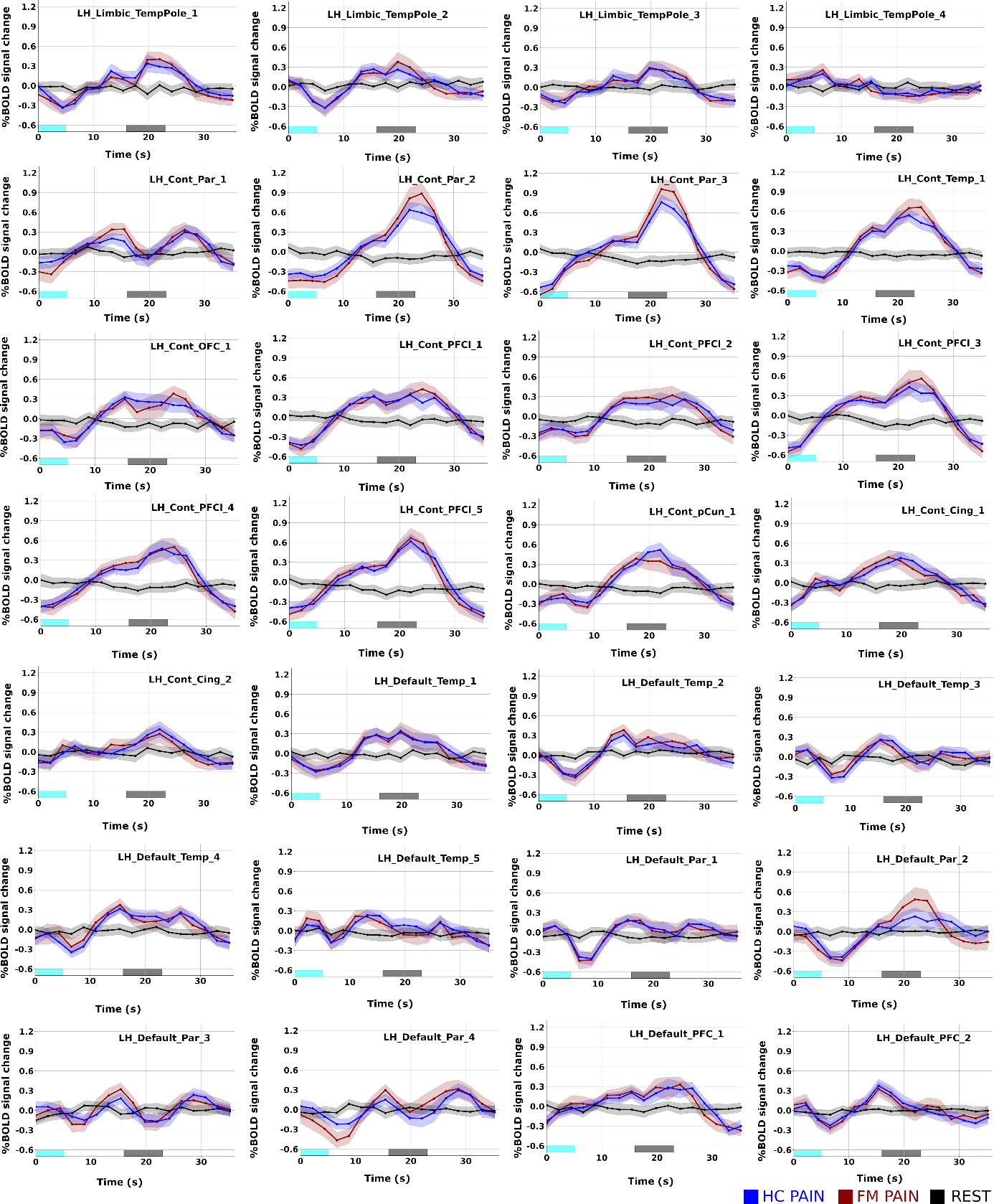
*

***Supplementary Figure 9 Cont****. Time-locked BOLD signal activation profiles for GM in the left hemisphere (LH).*


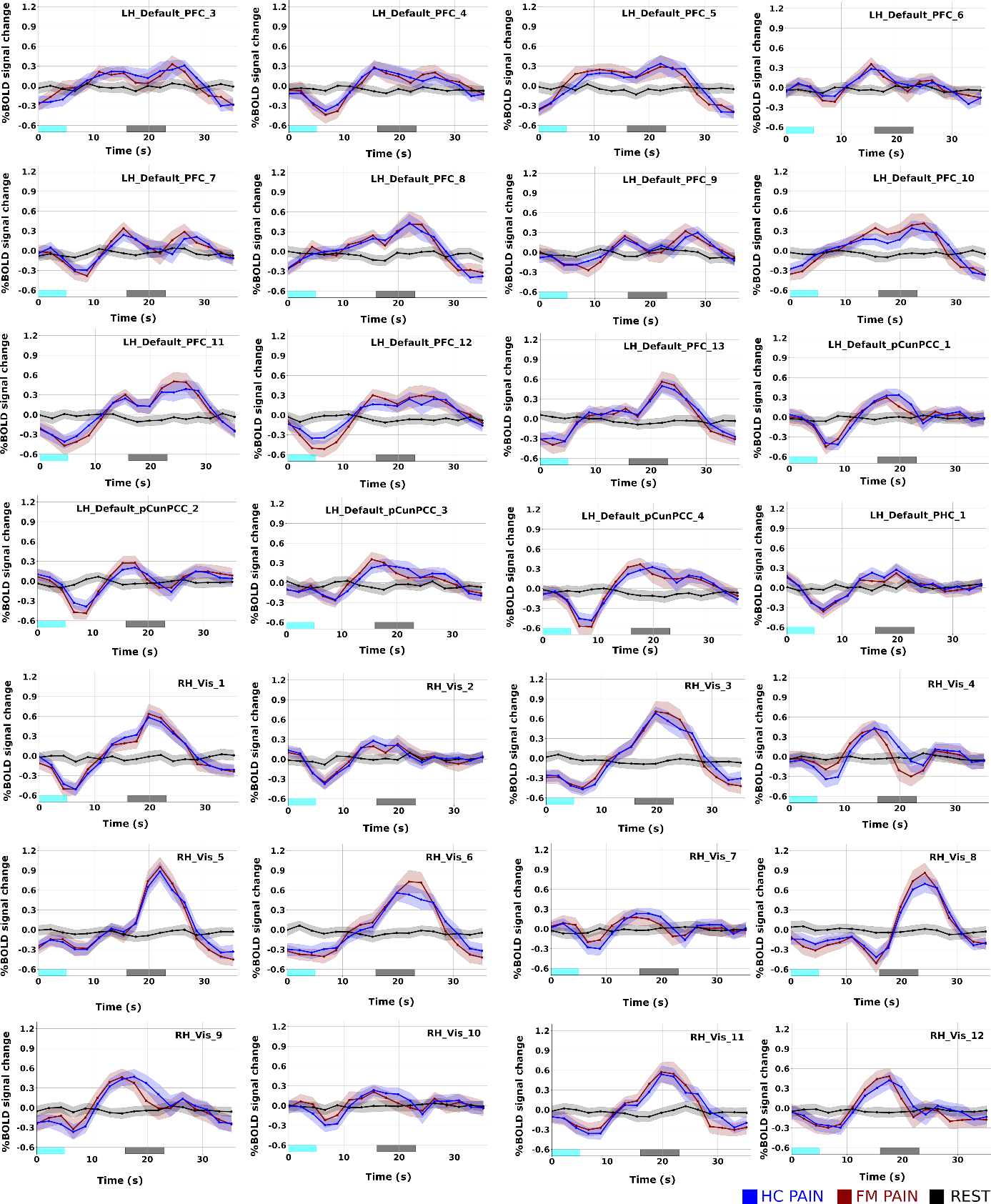


***Supplementary Figure 9 Cont****. Time-locked BOLD signal activation profiles for GM in the right hemisphere (RH) and left hemisphere (LR).*

*
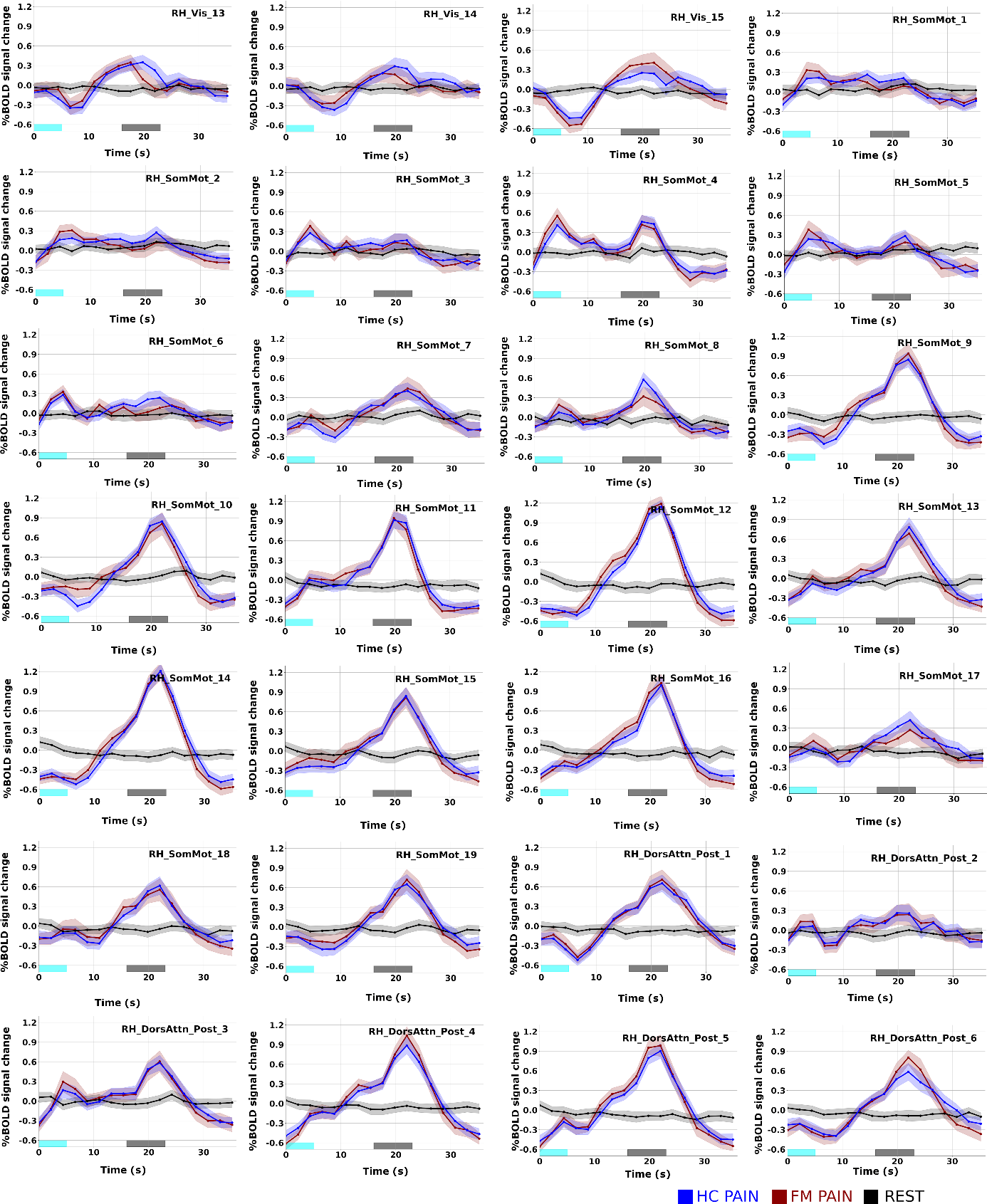
*

***Supplementary Figure 9 Cont****. Time-locked BOLD signal activation profiles for GM in the right hemisphere (RH).*

*
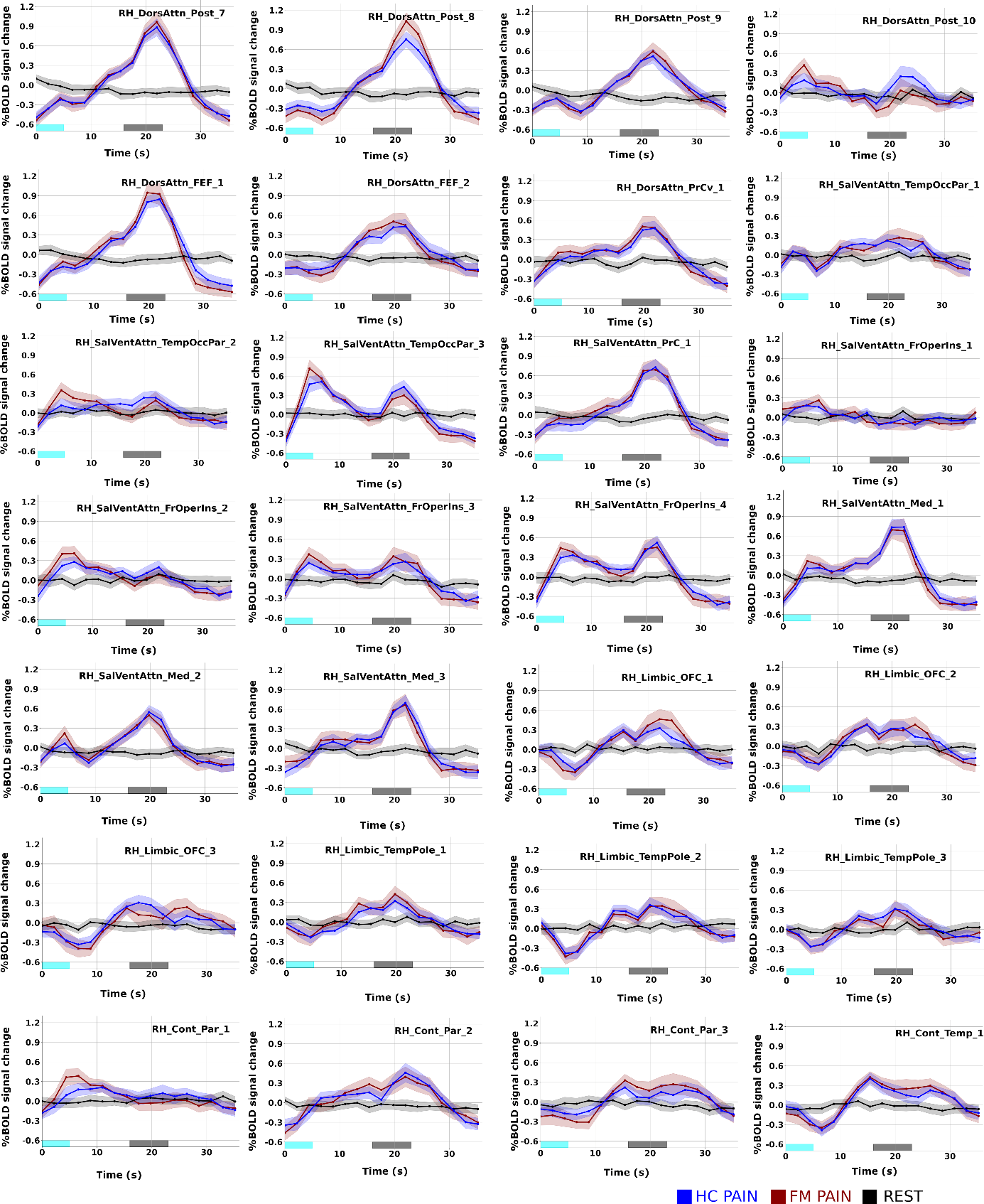
*

***Supplementary Figure 9 Cont****. Time-locked BOLD signal activation profiles for GM in the right hemisphere (RH).*

*
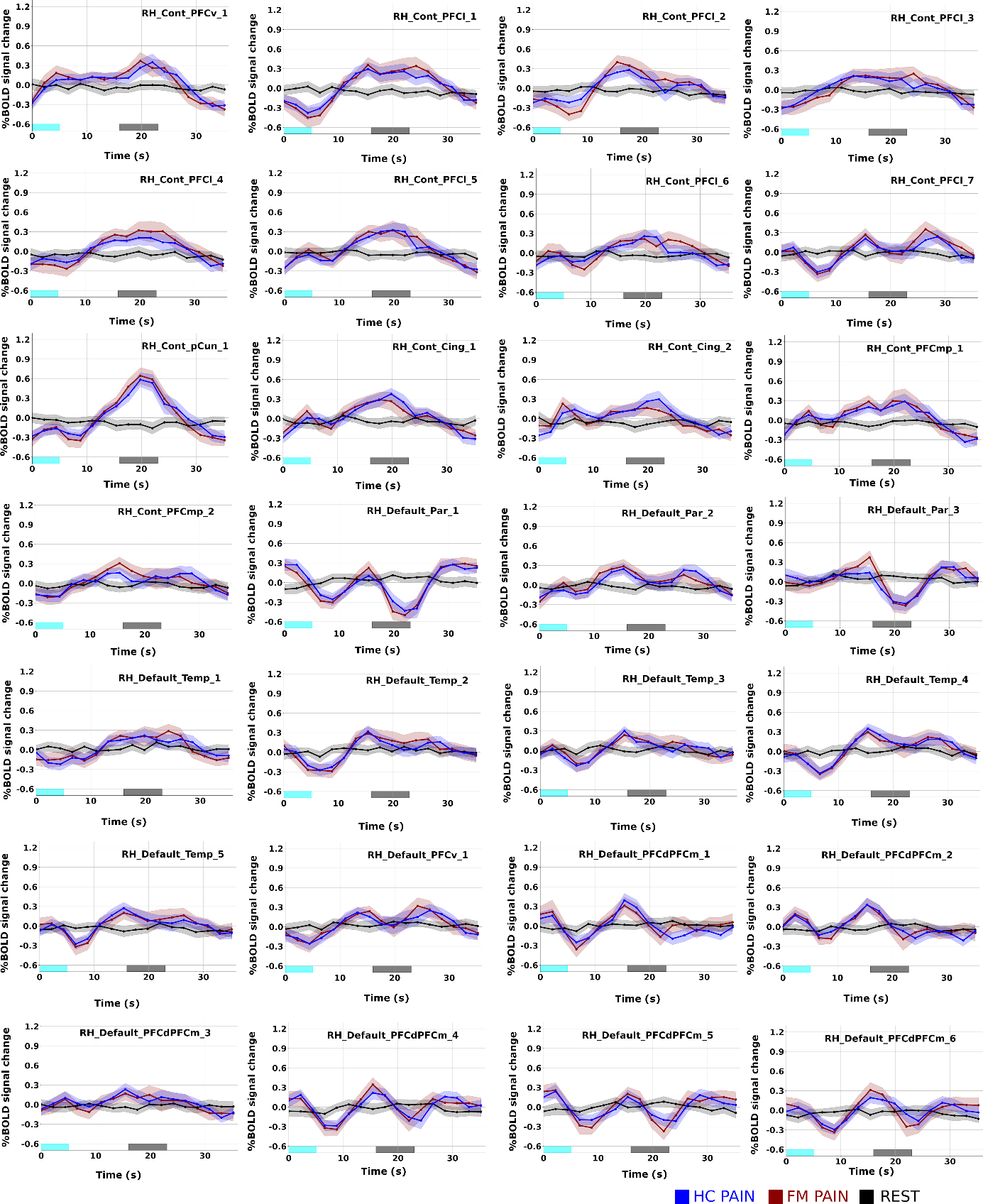
*

***Supplementary Figure 9 Cont****. Time-locked BOLD signal activation profiles for GM in the right hemisphere (RH).*

*
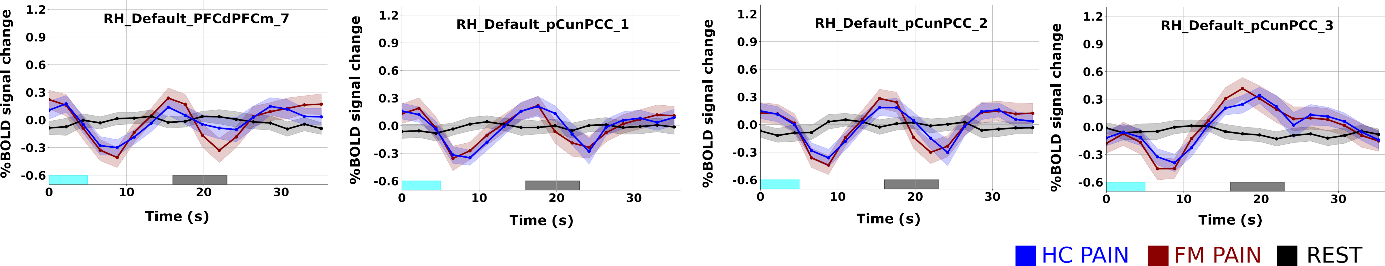
*

***Supplementary Figure 9 Cont****. Time-locked BOLD signal activation profiles for GM in the right hemisphere (RH).*

***Python code example for computing the periodogram for WM BOLD signal intensity timecourses in the healthy cohort and fibromyalgia participants:***

*#*

*# Example of a basic script to (1) compute the periodogram from WM BOLD signal timeseries in a*

*# blocked pain task paradigm, (2) extract the power amplitude at the experimental frequency*

*# (0.026 Hz) and (3) do a permutation test to test for significant differneces in power.*

*#*

*#!/usr/bin/env python3*

*import numpy as np*

*import matplotlib.pyplot as plt*

*import nilearn*

*from scipy.signal import periodogram*

*from scipy.stats import permutation_test*

*from statsmodels.stats.multitest import fdrcorrection*

*def statistic(x, y, axis):*

*return np.mean(x, axis=axis) - np.mean(y, axis=axis)*

*filepath1 = '/data/peter/fmigg/spm/subjects_preprocessed/sub-'*

*filepath2 = '/func/sub_'*

*filepath_WM_pain = '_task-ExpPain_run-2_bold_ICBM_WM_29ROIs.npy'*

*filepath_WM_spont = '_task-SponPain_bold_ICBM_WM_29ROIs.npy'*

*subj_pat_pain = [ '106', '107', '109', '112',*

*'115', '117', '118', '119', '121', '122', '123',*

*'126', '127', '134', '136', '138', '139',*

*'140', '143', '145', '147', '148', '151',*

*'152', '154', '156', '157', '159', '164', '165', '166',*

*'167', '168', '169', '170', '173', '175', '179', '182', '185', '186']*

*subj_cont_pain = ['301', '302', '303', '304', '309', '310', '311',*

*'312', '313', '314', '316', '317', '318', '320', '323', '324',*

*'325', '327', '329', '330', '331', '332', '335', '336',*

*'337', '338', '341', '342', '343', '346', '347', '348',*

*'349', '351', '352', '353', '354', '355', '356', '358',*

*'359', '361', '362', '363', '364', '365', '366', '367',*

*'369', '370', '374', '378', '379', '381' ]*

*T = 180*

*fs = 1 / 2.2*

*nrWM_ROIs = 29*

*nrSubj_pat_pain = 41*

*nrSubj_cont_pain = 54*

*nr_f_steps = 91*

*P_WM_pat_pain = np.zeros([nrSubj_pat_pain,nrWM_ROIs,nr_f_steps])*

*P_WM_cont_pain = np.zeros([nrSubj_cont_pain,nrWM_ROIs,nr_f_steps])*

*P10_WM_pat_pain = np.zeros([nrSubj_pat_pain, nrWM_ROIs])*

*P10_WM_cont_pain = np.zeros([nrSubj_cont_pain, nrWM_ROIs])*

*# read BOLD signal timecourses for WM regions in the FM pariticipant cohort.*

*for s in range(0,nrSubj_pat_pain) :*

*WM_tc_mtx = np.load(filepath1 + subj_pat_pain[s] + filepath2 + subj_pat_pain[s] + filepath_WM_pain)*

*for roi in range(0,nrWM_ROIs) :*

*# Compute the periodogram for each WM ROI and subject. Store the amplitude of power at the*

*# the experimental frequency in a separate matrix for later statistical tests.*

*[f, P] = periodogram(WM_tc_mtx[roi,:],fs, detrend = 'constant')*

*P_WM_pat_pain[s,roi,:] = P*

*P10_WM_pat_pain[s,roi] = P_WM_pat_pain[s,roi,10]*

*# read BOLD signal timecourses for WM regions in the HC cohort.*

*for s in range(0,nrSubj_cont_pain) :*

*WM_tc_mtx = np.load(filepath1 + subj_cont_pain[s] + filepath2 + subj_cont_pain[s] + filepath_WM_pain)*

*for roi in range(0,nrWM_ROIs) :*

*[f, P] = periodogram(WM_tc_mtx[roi,:],fs, detrend = 'constant')*

*P_WM_cont_pain[s,roi,:] = P*

*P10_WM_cont_pain[s,roi] = P_WM_cont_pain[s,roi,10]*

*# Do permutation tests.*

*p_values = np.zeros([nrWM_ROIs,])*

*for roi in range(0,nrWM_ROIs) :*

*res_stat = permutation_test((P10_WM_cont_pain, P10_WM_pat_pain), statistic, vectorized=True, n_resamples=10000, alternative='two-sided')*

*p_values[roi] = res_stat.pvalue*

*fdr_results = fdrcorrection(p_values, alpha=0.01, method='indep', is_sorted=False)*
